# Development of CRISPR/Cas12b-Based Multiple Cross Displacement Amplification Technique for the Detection of *Mycobacterium tuberculosis* Complex in Clinical Settings

**DOI:** 10.1128/spectrum.03475-22

**Published:** 2023-03-28

**Authors:** Xinggui Yang, Junfei Huang, Yijiang Chen, Xia Ying, Qinqin Tan, Xu Chen, Xiaoyan Zeng, Shiguang Lei, Yi Wang, Shijun Li

**Affiliations:** a Guizhou Provincial Center for Disease Control and Prevention, Guiyang, Guizhou, People’s Republic of China; b Second Affiliated Hospital, Guizhou University of Traditional Chinese Medicine, Guiyang, Guizhou, People’s Republic of China; c Experimental Research Center, Capital Institute of Pediatrics, Beijing, People’s Republic of China; University of Pretoria

**Keywords:** tuberculosis, *Mycobacterium tuberculosis* complex, CRISPR, Cas12b, multiple cross displacement amplification

## Abstract

Tuberculosis (TB) is a chronic infectious disease with high mortality caused by the Mycobacterium tuberculosis complex (MTC). Its clinical symptoms include a prolonged cough with mucus, pleuritic chest pain, hemoptysis, etc., and predominant complications such as tuberculous meningitis and pleural effusion. Thus, developing rapid, ultrasensitive, and highly specific detection techniques plays an important role in controlling TB. Here, we devised CRISPR/CRISPR-associated 12b nuclease (CRISPR/Cas12b)-based multiple cross displacement amplification technique (CRISPR-MCDA) targeting the *IS6110* sequence and used it to detect MTC pathogens. A newly engineered protospacer adjacent motif (PAM) site (TTTC) was modified in the linker region of the CP1 primer. In the CRISPR-MCDA system, the exponentially amplified MCDA amplicons with the PAM sites can guide the Cas12b/gRNA complex to quickly and accurately recognize its target regions, which successfully activates the CRISPR/Cas12b effector and enables ultrafast *trans*-cleavage of single-stranded DNA reporter molecules. The limit of detection of the CRISPR-MCDA assay was 5 fg/μL of genomic DNA extracted from the MTB reference strain H37Rv. The CRISPR-MCDA assay successfully detected all examined MTC strains and there was no cross-reaction with non-MTC pathogens, confirming that its specificity is 100%. The entire detection process can be completed within 70 min using real-time fluorescence analysis. Moreover, visualization detection (under UV light) was also designed to verify the results, eliminating the use of specialized instruments. In conclusion, the CRISPR-MCDA assay established in this report can be used as a valuable detection technique for MTC infection.

**IMPORTANCE** The Mycobacterium tuberculosis complex pathogen is a crucial infectious agent of tuberculosis. Hence, improving the capability of MTC detection is one of the most urgently required strategies for preventing and controlling TB. In this report, we successfully developed and implemented CRISPR/Cas12b-based multiple cross displacement amplification targeting the *IS6110* sequence to detect MTC pathogens. These results demonstrated that the CRISPR-MCDA assay developed in this study was a rapid, ultrasensitive, highly specific, and readily available method which can be used as a valuable diagnostic tool for MTC infection in clinical settings.

## INTRODUCTION

Tuberculosis (TB), a chronic infectious disease with high mortality, is caused by members of the Mycobacterium tuberculosis complex (MTC) and remains a serious public health problem globally (1, 2). This disease usually affects the lungs after infection with MTC organisms via air transmission (known as pulmonary TB), and its clinical symptoms develop slowly and are nonspecific (3, 4). Common symptoms include a prolonged cough lasting more than 2 weeks with mucus, pleuritic chest pain, hemoptysis, dyspnea, etc. (4, 5). Moreover, there are predominant complications associated with TB, such as tuberculous meningitis, tuberculous pleural effusion, miliary tuberculosis, and renal tuberculosis (4, 6, 7). In 2020, there were an estimated 10.0 million new TB cases and 1.5 million deaths (Global TB Report 2021) (8). In addition, in the background of the COVID-19 pandemic, HIV/TB co-infection was still present (HIV-positive patients account for 8% of new TB cases), making the prevention and treatment of TB increasingly tricky (8–10). Presently, MTC members mainly include Mycobacterium tuberculosis (MTB), M. bovis, M. africanum, M. caprae, and M. microti; among these, MTB infection accounts for the majority of TB patients (11, 12). As a result, rapid, ultrasensitive, and highly specific detection for MTC pathogens plays an important role in the treatment of TB as an effective strategy to control person-to-person transmission (13, 14).

Currently, the commonly used diagnostic tools for TB in clinical practice include traditional diagnostic methods (e.g., smear-staining microscopy and culture technique) and molecular detection techniques based on PCR (e.g., real-time quantitative PCR and GeneXpert MTB/RIF assays) (15–19). Despite the advantageous characteristics of traditional detection techniques, including visualization and reliability, these techniques are low-sensitive and time-consuming compared to molecular testing, and may lead to the risk of TB transmission (15, 19). Meanwhile, PCR-based techniques (namely, real-time PCR and GeneXpert MTB/RIF) may not be readily available due to their high demand for specialized amplification instruments (e.g., a thermocycler) and laboratory professionals (16, 20). Thus, there is an urgent need for an advanced, rapid, ultrasensitive, low-cost, and highly specific method for detecting MTC pathogens to achieve greater control of TB. Currently, various isothermal amplification techniques have been developed to further improve the sensitivity and cost of detection, as described in previous studies, such as recombinase polymerase amplification (RPA), loop-mediated isothermal amplification (LAMP), and multiple cross displacement amplification (MCDA) (21–23). In particular, the MCDA technique, an ultrasensitive and accessible point-of-care nucleic acid detection method, has been widely used to detect various pathogens, including viruses, bacteria, and fungi (24–26). In addition, the MCDA assay has broad application prospects because it requires only a single reaction enzyme (*Bst* DNA polymerase) in the exponential amplification process (27).

In recent years, the discovery of advanced CRISPR and CRISPR-associated protein (Cas) systems has been of landmark significance in the field of genome editing (28). Due to their rapid, intuitive, ultrasensitive, and highly specific characteristics, nucleic acid detection techniques based on CRISPR/Cas systems are regarded as next-generation molecular detection technologies (29). In the CRISPR/Cas system, the CRISPR/Cas effector proteins (e.g., Cas12a, Cas12b, Cas13a, and Cas13b) are specifically navigated by guide RNA (gRNA) to the target to form the complex (namely, target/Cas nuclease/gRNA complex), which can nonspecifically and indiscriminately cleave nearby nontarget single-stranded DNA (ssDNA) and/or ssRNA reporters when the *trans*-cleavage activity of Cas nuclease (i.e., collateral cleavage activity) is activated (29–34). Of the various Cas nucleases, the most widely used, Cas12a, Cas12b, and Cas13a, have been successfully combined with isothermal amplification techniques (33–35), and a series of CRISPR/Cas12a-, CRISPR/Cas12b-, and CRISPR/Cas13a-based nucleic acid detection techniques have been devised and used in past studies, such as DETECTR (DNA Endonuclease Targeted CRISPR *Trans* Reporter), SHERLOCK (Specific High Sensitivity Enzymatic Reporter UnLOCKing), and MCCD (multiple cross displacement amplification CRISPR/Cas12a detection) (29, 36, 37). These CRISPR/Cas-based assays greatly shorten detection time and improve detection sensitivity, providing an efficient diagnostic platform for target pathogen detection.

As described above, developing novel and ultrasensitive diagnostic techniques to improve TB testing capabilities will play a key role in future strategies to end TB, especially CRISPR/Cas system-based detection techniques. Subsequently, various molecular detection targets of MTC, including insertion sequence (IS) family (e.g., *IS6110* and *IS1081*), *mpb64*, *PstS1*, *mtp70*, and *gyrB* genes, have been successfully devised and used for laboratory detection of MTC (13, 16, 22, 38, 39). In particular, the *IS6110* sequence, a reliable and appropriate-length molecular target, has been extensively developed for rapid diagnostic assays, including RPA, LAMP, and MCDA assays (14, 22, 23, 34). Moreover, in a previous study, the CRISPR/Cas12b-gRNA system was activated and exhibited efficient cleavage activity after the PAM site guided the corresponding Cas12b/gRNA complex to its location (33). Here, the combination of the CRISPR/Cas12b system and MCDA technique targeting the *IS6110* sequence is a promising protocol.

Here, the CRISPR/Cas12b-based multiple cross displacement amplification technique (CRISPR-MCDA) targeting the *IS6110* sequence was devised and used for visual, reliable, ultrasensitive, and highly specific detection of MTC pathogens. In addition, a newly engineered PAM site (TTTC) was also modified in the linker region of the CP1 primer. In the CRISPR-MCDA system, the exponentially amplified MCDA amplicons with the PAM site can guide the Cas12b/gRNA complex to its target site and activate the CRISPR/Cas12b effector for *trans*-cleavage of the ssDNA reporter (5′-FAM-TTATTAT-BHQ1-3′). Next, the results were determined using real-time fluorescence analysis and visualization detection under UV light. The optimal reaction conditions and feasibility of the MTC-CRISPR-MCDA assay were confirmed using DNA templates extracted from reference strains, clinical isolates, and clinical sputum specimens.

## RESULTS

### The mechanism of the CRISPR-MCDA assay.

**FIG 1:**
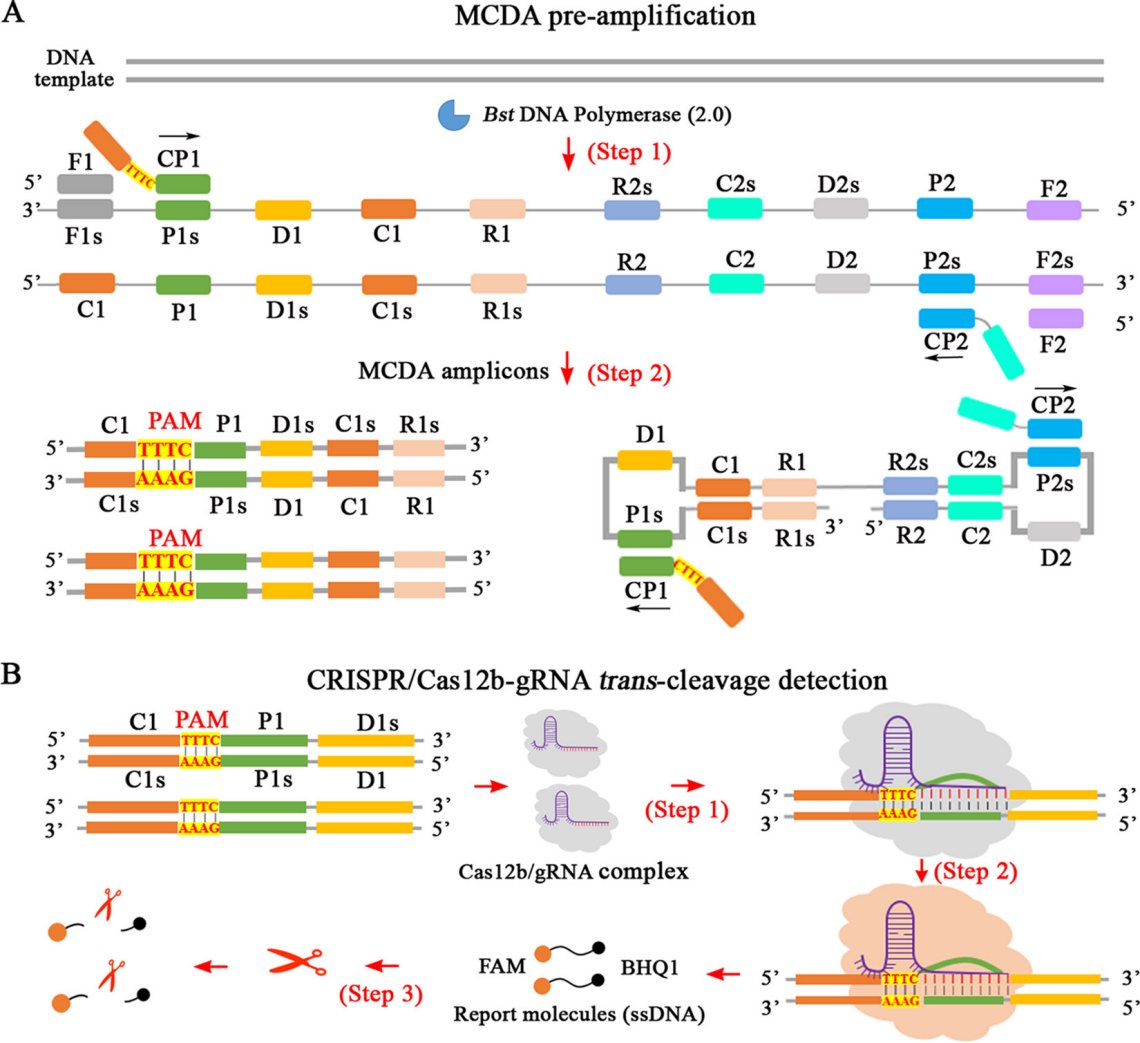
An overview of the reaction mechanism of the CRISPR/Cas12b-based multiple cross displacement amplification technique (CRISPR-MCDA) assay. (A) Description of the principle of MCDA pre-amplification. In the MCDA pre-amplification system, a new protospacer adjacent motif (PAM) site was specifically modified in the linker region of the CP1 primer (step 1). Then, the modified CP1 primer bound to the complementary region of the target and the MCDA products with the new PAM site were exponentially amplified, driven by the *Bst* DNA enzyme (step 2). (B) Mechanism of *trans*-cleavage of Cas12b/gRNA (guide RNA) complex. These MCDA products containing PAM sites guide the Cas12b/gRNA complex to recognize target sites (step 1). Subsequently, the *trans*-cleavage activity of the CRISPR/Cas12b system was activated to perform nonspecific digestion of ssDNA reporter probes (steps 2 and 3). As a result, the cleavage of ssDNA probe that releases BHQ1 allows the fluorophore to fluoresce (step 3). MTB, Mycobacterium tuberculosis.

**FIG 2:**
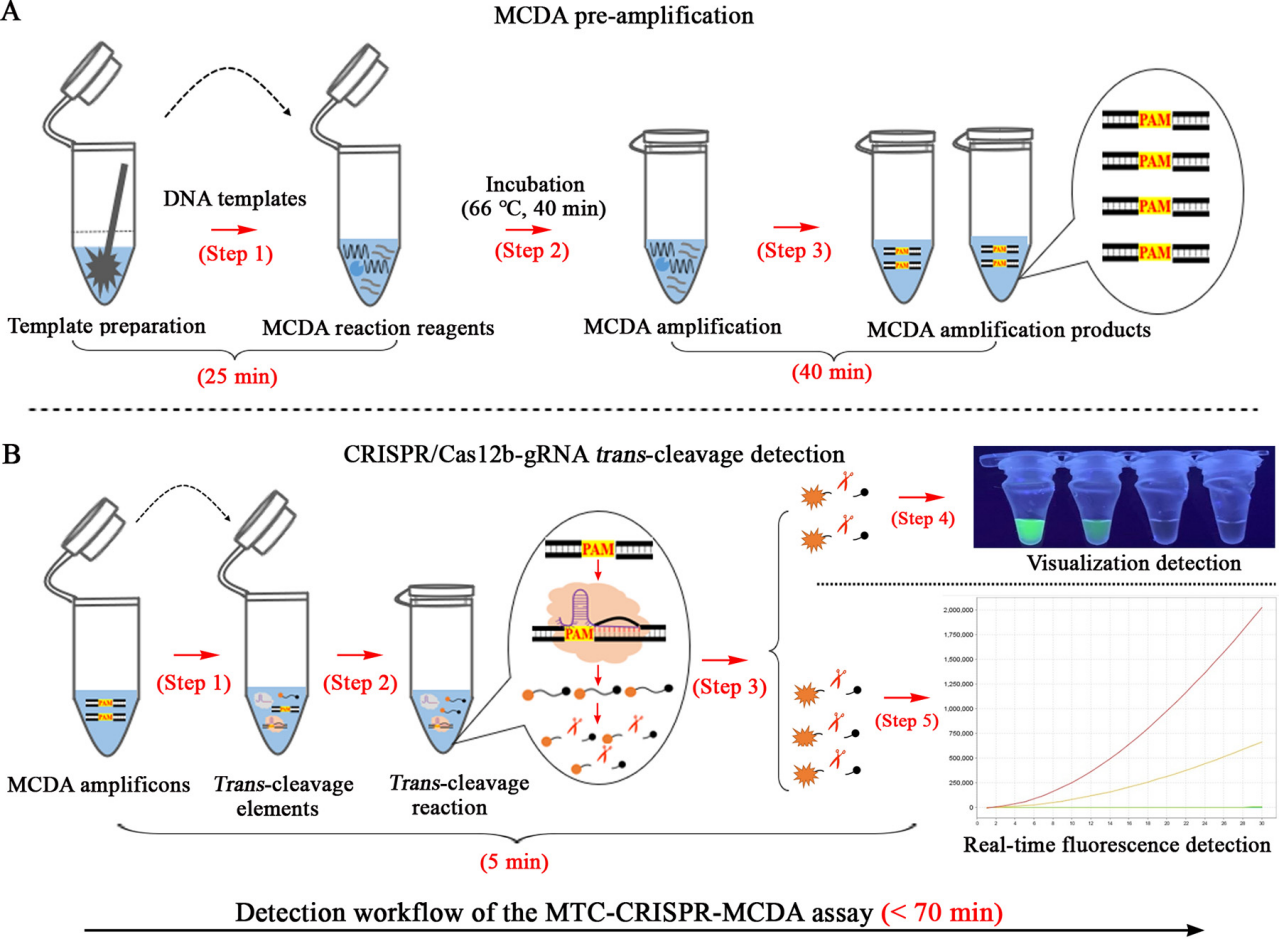
Workflow of the CRISPR-MCDA assay. (A) Workflow of MCDA pre-amplification. DNA templates prepared using the rapid extraction method were premixed with MCDA amplification reagents and specific primers (step 1). The premixed reaction system was incubated at 66°C for 40 min (step 2), and then the MCDA amplicons with the PAM site were exponentially amplified (step 3). (B) Workflow of CRISPR/Cas12b-gRNA-mediated *trans*-cleavage detection. Two μL of MCDA pre-amplification products was added to the pre-mixed system containing the Cas12b/gRNA complex (step 1), which was able to recognize the target site under the guidance of the PAM site, resulting in activation of the CRISPR/Cas12b effector (step 2). The single-stranded DNA (ssDNA) reporters were cleaved and subsequently released BHQ1, which causes the fluorophore to fluoresce (step 3). These emitted fluorescent signals were detected by visual detection under UV light (step 4) or using a real-time fluorescence detector (step 5).

The reaction mechanism of the whole CRISPR-MCDA assay is shown in Fig. 1 and 2, and was mainly composed of MCDA pre-amplification (Fig. 1A and 2A, steps 1 and 2) and CRISPR/Cas12b-mediated *trans*-cleavage detection (Fig. 1B and 2B). In the CRISPR-MCDA system, the CP1 primer with a PAM site (TTTC) modified in the linker region was specifically constructed according to the principle of Cas12b/gRNA effect (Fig. S1, Table S1). Due to the above design, the amplicons containing a newly prerequisite PAM site (TTTC) were amplified exponentially in MCDA amplification catalyzed by the *Bst* enzyme (Fig. 1A, steps 1 and 2), which can be used to guide the pre-assembled Cas12b/gRNA system to the target site (Fig. 1B, step 1). Next, the *trans*-cleavage activity was activated upon binding of the CRISPR/Cas12b-gRNA system to the guide-complementary MCDA products (Fig. 1B, step 2), resulting in cleavage of the ssDNA reporter probe which was specifically labeled fluorophore (6-carboxyfluorescein, FAM) and quencher (BHQ1) at the 5′ and 3′ ends, respectively (5′-FAM-TTATTAT-BHQ1-3′) (Fig. 1B, step 3). As a result, the cleavage of the ssDNA reporter that releases BHQ1 when the *trans*-cleavage activity of the CRISPR/Cas12b system is activated allows the fluorophore to fluoresce (Fig. 2B, steps 1, 2, and 3). The resulting fluorescence signal can then be detected by visualization under UV light (Fig. 2B, step 4) and a real-time fluorescence detector (Fig. 2B, step 5). Due to the high *trans*-cleavage activity of CRISPR/Cas12b system, the cutting process of ssDNA reporters released sufficient fluorescence signal for MTC detection in a short time (approximately 5 min).

### Confirmation test of CRISPR-MCDA assay.

First, we verified the feasibility of the CRISPR-MCDA assay by a confirmation test. The CRISPR-MCDA assay was tested using one microliter (1 ng/μL) of genomic DNA of MTB H37Rv as the template according to the reaction conditions described. In our study, MCDA pre-amplification results were detected using a real-time turbidimeter (Fig. S2A), an MG visual indicator (Fig. S2B), and 1.5% agarose gel electrophoresis (Fig. S2C). Then, as shown in Fig. S2, the ssDNA reporter molecules were cleaved in the Cas12b/gRNA-mediated detection, and then the sufficient fluorescence signals released in this system were captured by a real-time fluorescence detector (Fig. S2D) or visualized under UV light (Fig. S2E).

### Optimal pre-amplification conditions of CRISPR-MCDA assay.

To explore the optimal reaction conditions (i.e., pre-amplification temperature and time), the optimization tests were performed. In the temperature optimization test shown in Fig. S3, eight dynamic curves showed efficient and robust amplification at reaction temperatures ranging from 65 to 68°C (Fig. S3D to G). The MCDA pre-amplification products were also detected by Cas12b/gRNA-mediated detection, and the fluorescence signal released at 66°C was stronger than that released at other temperatures as determined by real-time fluorescence analysis (Fig. S4E). In addition, the lowest concentration (5 fg/μL) of serial dilutions of MTB genomic DNA that could be detected by the CRISPR-MCDA assay was consistent with a reaction time of 40 to 50 min (Fig. S5 and S6). Importantly, the verification results were also consistent when CRISPR/Cas12b-mediated *trans*-cleavages were validated by real-time fluorescence detection (Fig. S5D to F) and visualization under UV light (Fig. S6D to F). Thus, the optimal amplification conditions for CRISPR-MCDA assay established in this report was 66°C for 40 min (Table 1).

**TABLE 1:** Optimization of pre-amplification time for the CRISPR-MCDA assay^*a*^

Time (min)	Serial dilutions of genomic DNA (MTB H37Rv) (per μL)									
	1 ng	100 pg	10 pg	1 pg	100 fg	10 fg	5 fg	1 fg	500 ag	BC
25	+/+	+/+	+/+	+/−	−/−	−/−	−/−	−/−	−/−	−/−
30	+/+	+/+	+/+	+/+	+/−	−/−	−/−	−/−	−/−	−/−
35	+/+	+/+	+/+	+/+	+/+	+/+	−/−	−/−	−/−	−/−
40	+/+	+/+	+/+	+/+	+/+	+/+	+/+	−/−	−/−	−/−
45	+/+	+/+	+/+	+/+	+/+	+/+	+/+	−/−	−/−	−/−
50	+/+	+/+	+/+	+/+	+/+	+/+	+/+	−/−	−/−	−/−

a Values given as fluorescence signal (positive/negative)/visualization (positive/negative). BC, blank control.

### Optimal *trans*-cleavage time of CRISPR-MCDA assay.

When the CRISPR/Cas12b cleavage time ranged from 5 to 30 min, as displayed in Fig. S7, the positive and negative reactions could be accurately differentiated, and the lowest concentration of genomic DNA (MTB H37Rv) detectable by real-time fluorescence analysis was 5 fg/μL (Fig. S7A to F). Furthermore, we also explored the optimal visualization time of the Cas12/gRNA-mediated *trans*-cleavage test in our study. When the cleavage time was 5 to 20 min, it was difficult to accurately distinguish between positive and negative reactions by visualization under UV light despite the strong fluorescence signals detected using a real-time fluorescence detector (Fig. S8A, B, C, and D). The results could be determined only with a cutting time of 25 to 30 min (Fig. S8E and F). In particular, the lowest concentration of MTB genomic DNA that was accurately detectable by visualization was 5 fg/μL when the cleavage time was 30 min (Fig. S8F). As a result, the optimal *trans*-cleavage time for the CRISPR-MCDA assay developed in our study was 5 min by real-time fluorescence analysis and/or 30 min by visual detection under UV light (Table 2).

**TABLE 2:** Optimization of *trans*-cleavage time for the CRISPR-MCDA assay^*a*^

Time (min)	Serial dilutions of genomic DNA (MTB H37Rv) (per μL)									
	1 ng	100 pg	10 pg	1 pg	100 fg	10 fg	5 fg	1 fg	500 ag	BC
5	+/−	+/−	+/−	+/−	+/−	+/−	+/−	−/−	−/−	−/−
10	+/−	+/−	+/−	+/−	+/−	+/−	+/−	−/−	−/−	−/−
15	+/+	+/+	+/+	+/+	+/−	+/−	+/−	−/−	−/−	−/−
20	+/+	+/+	+/+	+/+	+/−	+/−	+/−	−/−	−/−	−/−
25	+/+	+/+	+/+	+/+	+/+	+/+	+/+^*b*^	−/−	−/−	−/−
30	+/+	+/+	+/+	+/+	+/+	+/+	+/+	−/−	−/−	−/−

a Values given as fluorescence signal (positive/negative)/visualization (positive/negative). BC, blank control.

b Fluorescence signal (positive)/visualization (weak positive).

### Analytical sensitivity of MTC-CRISPR-MCDA assay.

**FIG 3:**
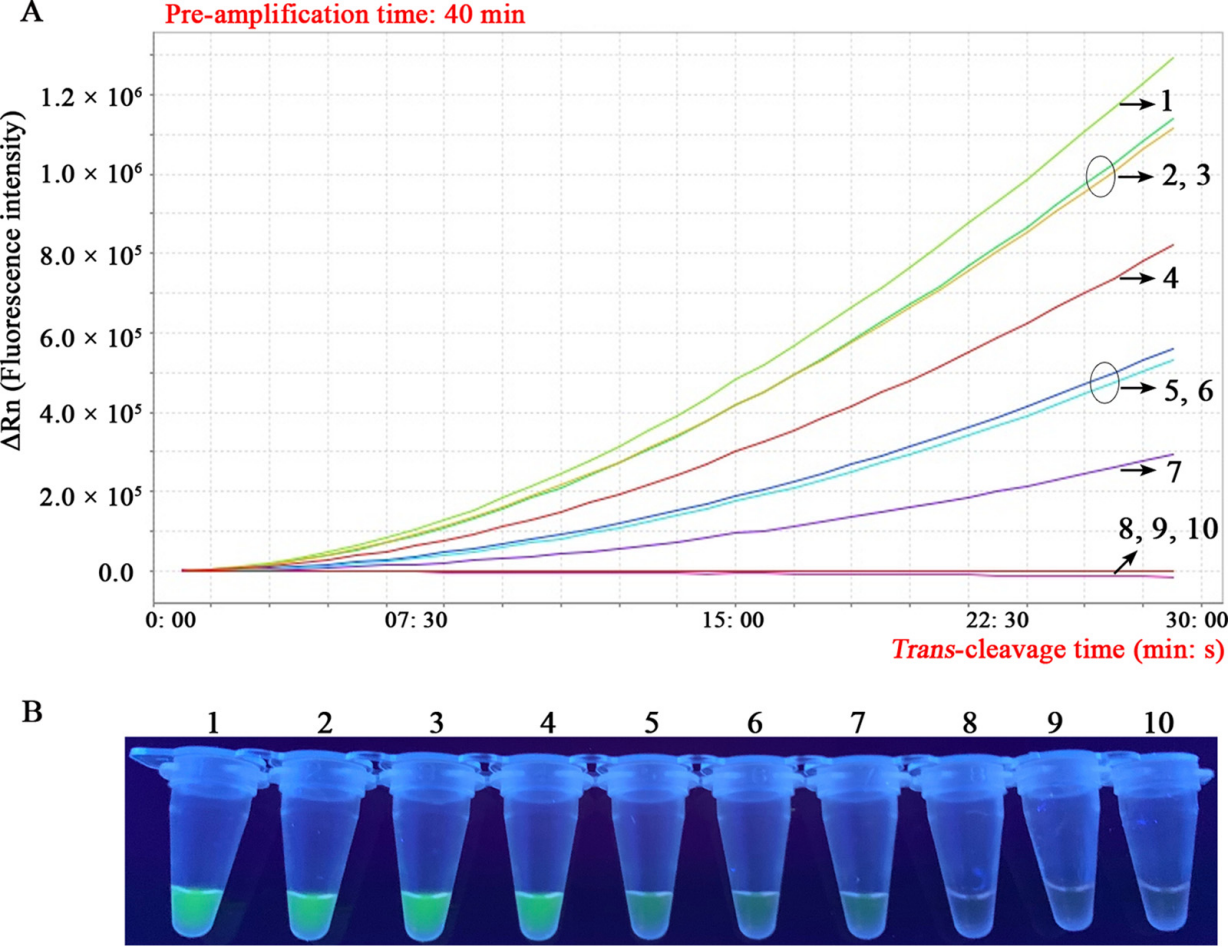
Analytical sensitivity of the CRISPR-MCDA assay. (A) Real-time fluorescence analysis was used to report the results of CRISPR-MCDA assays. (B) CRISPR-MCDA results were detected by visualization under UV light. One μL of each dilution of MTB H37Rv genome (1 ng/μL, 100 pg/μL, 10 pg/μL, 1 pg/μL, 100 fg/μL, 10 fg/μL, 5 fg/μL, 1 fg/μL, and 500 ag/μL) was used as the templates. Signals (A)/tubes (B) 1 to 9 correspond to DNA template of MTB H37Rv from 1 ng/μL to 500 ag/μL. Signal (A)/tube (B) 10 corresponds to blank control (nuclease-free water). The lowest concentration (5 fg/μL) of serial dilution of MTB genomic DNA that was detectable by real-time fluorescence and visualization detection was consistent (panel A, line 7; and panel B, lane 7).

To evaluate the analytical sensitivity of the CRISPR-MCDA assay, we tested and analyzed its limit of detection (LoD) according to the specified definitions. As shown in Fig. 3, the CRISPR-MCDA assay LoD was 5 fg/μL, and the results of real-time fluorescence analysis (Fig. 3A) and visualization detection (under UV light) (Fig. 3B) were consistent. Importantly, the analytical sensitivity of the CRISPR-MCDA assay was 200-fold higher than that of the MTC-PCR assay, with a LoD of 1 pg/μL (Fig. 3 and Fig. S9), and 20-fold higher than that of the MTC-LAMP assay, with a LoD of 100 fg/μL (Fig. 3 and S10).

### Analytical specificity of MTC-CRISPR-MCDA assay.

**FIG 4:**
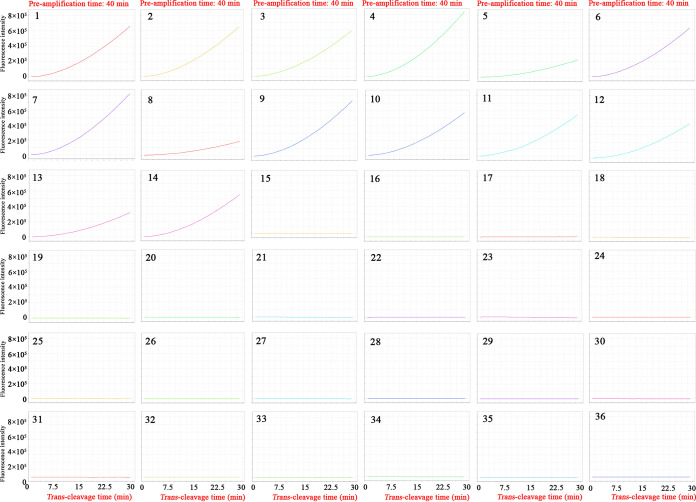
Analytical specificity of the CRISPR-MCDA assay using real-time fluorescence analysis. The CRISPR-MCDA assay was performed using DNA templates extracted from various pathogens, and the results were validated by real-time fluorescence detection. Signals 1 to 2: MTB H37Rv (ATCC 27294) and MTB H37Ra (ATCC 25177). Signals 3 to 11: MTB isolates (GZCDC). Signals 12 to 14: Mycobacterium bovis (ATCC 19210), M. bovis bacillus Calmette-Guérin vaccine strain, and M. africanum (ATCC 25420). Signals 15 to 25: M. smegmatis (ATCC 19420), M. nonchromogenicum (ATCC 19530), M. aichiense (ATCC 27280), M. neoaurum (ATCC 27280), M. neoaurum (ATCC 25795), M. scrofulaceum (ATCC 19981), M. xenopi (ATCC 19250), M. ulcerans (ATCC 19423), M. malmoense (ATCC 29571), M. abscessus (ATCC 19977), M. kansassi (ATCC 12478), and M. vaccae (ATCC 15483). Signals 26 to 35: Brucella melitensis M5 (vaccine strain), Klebsiella pneumoniae (GZCDC), Pseudomonas aeruginosa (GZCDC), Haemophilus influenzae (GZCDC), Streptococcus pneumoniae (GZCDC), Staphylococcus aureus (GZCDC), Shigella sonnei (GZCDC), Bacillus anthracis (GZCDC), Streptococcus suis (GZCDC), and Salmonella species strains (GZCDC). Signal 36: blank control (nuclease-free water). GZCDC, Guizhou Provincial Center for Disease Control and Prevention.

**FIG 5:**
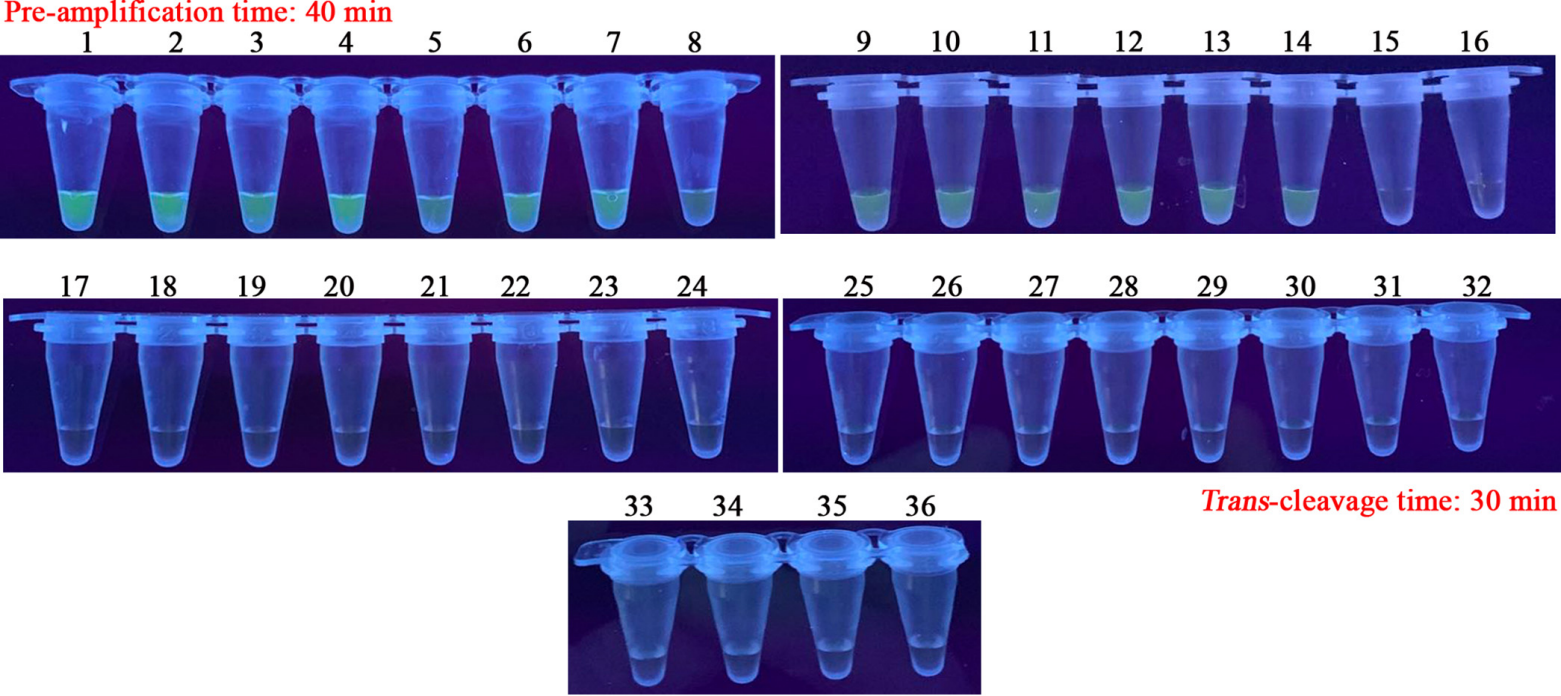
Analytical specificity for the CRISPR-MCDA assay using visualization analysis. The CRISPR-MCDA assay was performed using DNA templates extracted from various pathogens, and the results were validated by visual detection under UV light. Tubes 1 and 2: MTB H37Rv (ATCC 27294) and MTB H37Ra (ATCC 25177). Tubes 3 to 11: MTB isolates (GZCDC). Tubes 12 to 14: M. bovis (ATCC 19210), M. bovis bacillus Calmette-Guérin vaccine strain, and M. africanum (ATCC 25420). Tubes 15 to 25: M. smegmatis (ATCC 19420), M. nonchromogenicum (ATCC 19530), M. aichiense (ATCC 27280), M. neoaurum (ATCC 27280), M. neoaurum (ATCC 25795), M. scrofulaceum (ATCC 19981), M. xenopi (ATCC 19250), M. ulcerans (ATCC 19423), M. malmoense (ATCC 29571), M. abscessus (ATCC 19977), M. kansassi (ATCC 12478), and M. vaccae (ATCC 15483). Tubes 26 to 35: B. melitensis M5 (vaccine strain), K. pneumoniae (GZCDC), P. aeruginosa (GZCDC), H. influenzae (GZCDC), S. pneumoniae (GZCDC), S. aureus (GZCDC), S. sonnei (GZCDC), B. anthracis (GZCDC), S. suis (GZCDC), and Salmonella species strains (GZCDC). Tube 36: blank control (nuclease-free water).

In the current report, the analytical specificity of CRISPR-MCDA assay was evaluated using DNA templates extracted from various pathogens, including representative MTC, non-tuberculous mycobacteria (NTM), and non-mycobacteria strains. The analytical specificity of CRISPR-MCDA test established in the experiment was 100%, and it was able to detect the DNA templates of representative MTC strains (including MTB H37Rv, MTB H37Ra, M. bovis, M. bovis bacillus Calmette-Guérin vaccine strain, M. africanum, and 9 MTB isolates) and exclude the genomic DNA of NTM strains and non-mycobacteria strains (Fig. 4 and 5, Table S3).

### Practicability of CRISPR-MCDA for testing clinical sputum specimens.

A total of 90 clinical specimens were examined by conventional methods (smear staining microscopy and mycobacterial culture) and molecular detection assays (i.e., MTC-CRISPR-MCDA, -PCR, and -LAMP assays). Here, for the test results of sputum samples from TB patients, 36 sputum samples tested positive and 27 tested negative by smear-staining microscopy (57.14%, 36/63); Forty-six specimens were tested positive and 17 tested negative by the culture method (73.02%, 46/63). In the MTC-PCR test, 46 clinical samples tested positive and 17 tested negative (73.02%, 46/63); Forty-eight sputum specimens tested positive and 15 tested negative via MTC-LAMP assay (76.19%, 48/63); Fifty-one specimens tested positive and 12 tested negative by MTC-CRISPR-MCDA assay (80.95%, 51/63) (Table 3). Moreover, 27 sputum specimens collected from non-TB patients tested negative by the five methods above (Table 3). As described above, the CRISPR-MCDA assay established in this study possessed higher sensitivity in sputum samples than the other four methods and can be used as a potential tool for diagnosing TB in clinical settings.

**TABLE 3:** Comparison of five detection methods for clinical sputum samples^*a*^

Method	Patient sample origin		Sensitivity (%)	Specificity (%)	PPV (%)^*b*^	NPV (%)
	TB (*n* = 63)	non-TB (*n* = 27)				
SSM						
Positive	36	0	57.14	100	100	50.00
Negative	27	27				
Culture						
Positive	46	0	73.02	100	100	61.36
Negative	17	27				
MTC-PCR						
Positive	46	0	73.02	100	100	61.36
Negative	17	27				
MTC-LAMP						
Positive	48	0	76.19	100	100	64.29
Negative	15	27				
CRISPR-MCDA						
Positive	51	0	80.95	100	100	69.23
Negative	12	27				

a SSM, sputum smear microscopy; LAMP, loop-mediated isothermal amplification; MCDA, multiple cross displacement amplification; TB, tuberculosis; PPV, positive predictive value; NPV, negative predictive value.

b PPV = (true positive/[true positive + false positive]) × 100; NPV = (true negative/[true negative + false negative]) × 100.

## DISCUSSION

To date, the MTC pathogen, a crucial infectious agent of TB, cannot be ignored because of its high infectivity and pathogenicity, especially in areas with insufficient detection capability (40, 41). Hence, improving the capability of MTC detection is one of the most urgently required strategies for controlling TB, including the ongoing development and improvement of rapid, reliable, sensitive, and readily available diagnostic techniques. Although smear-staining microscopy and culture remain the conventional methods for diagnosing TB, their inherent time-consumption, low sensitivity, and risk of infection for laboratory operators make it challenging to meet the previously mentioned requirements (15, 19). Admittedly, these rapid detection techniques (including PCR and PCR-based assays and immunoassay-based T-SPOT and enzyme-linked immunosorbent assay [ELISA]) are conventionally used in MTC tests with certain advantages (16, 42, 43). Still, their deficiencies (low sensitivity, high detection cost, cumbersome operation process, and requirement of special detectors) hinder their further application in clinical examination (16, 43). An ideal diagnostic tool would be fast, sensitive, specific, low-cost, and easy to use to improve the capability to diagnose TB.

Currently, CRISPR/Cas system-based techniques, known as next-generation nucleic acid detection strategies, have significant development and application value in the field of molecular diagnostics (29). Among these, CRISPR/Cas systems such as CRISPR/Cas12a, CRISPR/Cas12b, and CRISPR/13a have been widely used for the detection of various pathogens (33, 34, 36). Meanwhile, the Cas12b nuclease, a newly developed sequence-specific effector, has been successfully designed for genome editing in many fields and has highly efficient *trans*-cleavage activity at an optimal temperature of approximately 48°C (33, 44). Currently, CRISPR/Cas-based isothermal techniques are a research focus in this field, as they do not require a dedicated apparatus (in particular, isothermal conditions only) (29, 35). The MCDA technique, an attractive nucleic acid detection test, was designed and developed, showing high sensitivity and specificity because its primers can recognize the 10 regions of the target (Fig. 1, step 1) (27, 29, 33). As a result, the CRISPR-MCDA assay, which combines MCDA pre-amplification with the Cas12b/gRNA system, was established and used for efficient detection of MTC pathogens in this study. Moreover, the CRISPR/Cas system effectively overcame the limitation of a target sequence not containing a PAM site because the engineered PAM site (TTTC) was designed in the linker region of the CP1 primer. This strategy ensured that Cas12b effectors were activated after the PAM sites guided the corresponding Cas12b/gRNA complex to its location, resulting in the ssDNA probe (5′-FAM-TTATTAT-BHQ1-3′) being rapidly digested in a short time and then releasing substantial fluorescence signals. In other words, the CRISPR-MCDA assay can be used for the detection of other nucleic acids if the MCDA primer design principles are met.

In the CRISPR-MCDA system, real-time fluorescence analysis was used to verify the detection results, which only took about 5 min (Fig. 2B, step 5). In fact, when the *trans*-cleavage time was set to 5 min, the time required to determine the detection result was only within 4 min by the optimization test of Cas12b/gRNA-mediated real-time fluorescence analysis (Fig. S7A). As a result, the entire detection process, including DNA template preparation (25 min), MCDA pre-amplification (40 min), and Cas12b/gRNA-mediated *trans*-cleavage of ssDNA reporter (5 min), can be completed within 70 min (Fig. 2, Table S2). Currently, many CRISPR/Cas-mediated nucleic acid detection techniques rely on a lateral flow biosensor to verify their amplicons, which increases the test’s cost and/or operational complexity (29, 33). Meanwhile, a similar CRISPR/Cas12b-mediated LAMP technique has been used in past studies to detect MTB pathogens. However, this method is time-consuming (approximately 2 h) and its extreme reliance on real-time fluorescence system makes it not readily available for the rapid detection of MTB pathogens (45). Hence, the visualization detection was designed and performed in our study, and the detection results can be directly determined by the naked eye under UV light (Fig. 2B, step 4). Although the optimal reaction time for visual validation was 30 min, the entire examination procedure can be performed with a simple thermostatic device (even a thermostatic water bath). As mentioned above, the CRISPR-MCDA assay, as an attractive detection tool, has potential application value for the clinical diagnosis of TB.

The *IS6110* sequence, a common molecular target for MTC detection in previous studies, was used to design the specific MCDA primers and gRNA in the CRISPR-MCDA system (14, 34). Next, the optimal MCDA pre-amplification conditions and Cas12b/gRNA-mediated *trans*-cleavage time were confirmed by optimization assays, successfully establishing the MTC-CRISPR-MCDA assay in the current report (Fig. S5, S6, S7, and S8). The CRISPR-MCDA assay demonstrated high analytical sensitivity and was able to detect MTB genomic DNA at the lowest concentration of 5 fg/μL in this report. Moreover, real-time fluorescence and visualization detection were consistent for the validation of Cas12b/gRNA-mediated *trans*-cleavage results (Fig. 3). Importantly, the analytical sensitivity of CRISPR-MCDA was 20-folds higher than that of the MTC-LAMP assay (Fig. S10, Table S2) and at least 200-folds higher than that of the MTC-PCR assay (Fig. S9, Table S2), which is essential for improving the detection rate of potential MTC infection. In addition, the analytical sensitivity of the CRISPR-MCDA assay (5 fg/μL) is higher than that of the similar MTC-CRISPR-LAMP (50 fg/μL) and MTC-MCDA-LFB assays (100 fg/μL) (22, 34).

In the specificity assays, there was no cross-reaction, and the CRISPR-MCDA test established in the study was able to detect all representative MTC strains (including MTB H37Rv, MTB H37Ra, M. bovis, M. bovis bacillus Calmette-Guérin vaccine strain, M. africanum, and 9 MTB isolates) and exclude 12 NTM and non-mycobacteria strains (Fig. 4 and 5). The CRISPR-MCDA assay showed high specificity for recognizing MTC pathogens, which provides methodological support for accurately diagnosing TB in clinical practice. Undeniably, MCDA pre-amplification is prone to a high concentration of amplicons due to its high sensitivity, resulting in carryover contamination (26). The CRISPR/Cas system can effectively avoid residual contamination by cutting the MCDA amplicons, ensuring the reliability of CRISPR-MCDA tests (29, 32). Furthermore, the test should be strictly performed in different areas, including DNA template preparation, reaction system pre-mixing, MCDA pre-amplification, and *trans*-cleavage detection (46).

Moreover, the CRISPR-MCDA assay established in this study had a higher sensitivity (80.95%, 51/63) for detection from sputum samples than staining microscopy, culture, MTC-PCR, and MTC-LAMP assays (Table 3 and Table S2). Importantly, the clinical samples which tested positive by smear staining microscopy, culture, MTC-PCR, and MTC-LAMP also tested positive by MTC-CRISPR-MCDA assay. These data indicate that the CRISPR-MCDA assay developed in this study was suitable for testing clinical sputum specimens and can be used as a valuable screening or diagnostic tool for TB. However, 12 clinical samples collected from TB patients, the positive group, tested negative by CRISPR-MCDA assay, possibly due to the following reasons: (i) incorrect sampling methods and/or limitations of sample quality (e.g., impurities); (ii) too few bacteria, resulting in a low concentration of the prepared DNA template (less than 5 fg/μL); or (iii) a lack of the *IS6110* sequence in the bacterial genomes of these samples (3, 14). Previously and currently, the culture method has usually been used as the gold standard for TB diagnosis, but its preponderances (e.g., positive rate, detection time, and operation procedures) were not apparent compared with the CRISPR-MCDA method developed in our study (3, 34). In conclusion, the CRISPR-MCDA assay has enormous potential for the clinical diagnosis of TB.

In this report, the total cost of a single CRISPR-MCDA reaction for the clinical sample did not exceed 4.5 US$, including MCDA isothermal reagent (approximately 1.5 US$ per reaction), AapCas12b nuclease (approximately 1.5 US$ per reaction), and other reagents and materials (approximately 1.5 US$ per reaction). Meanwhile, real-time fluorescence analysis greatly shortened the time required for the test workflow, resulting in the detection process being completed within 70 min. Furthermore, the visualization detection method developed in the CRISPR-MCDA assay eliminated the requirement for specialized instruments (e.g., PCR thermal cycler) and saved the cost of the LFB biosensors used in previous studies. Overall, the CRISPR-MCDA assay developed in this study is a convenient, reliable, ultrasensitive, and readily available diagnostic tool for MTC infection in clinical settings.

In conclusion, in this report, the CRISPR-MCDA technique targeting the *IS6110* sequence was newly designed and established for the detection of MTC pathogens. The MTC-CRISPR-MCDA assay established here demonstrated high sensitivity and specificity for testing pure cultures and clinical sputum samples. Real-time fluorescence analysis and visualization detection were used to validate the CRISPR-MCDA reaction results, resulting in greatly shortened examination time and eliminating the use of dedicated instruments. Hence, the MTC-CRISPR-MCDA assay established here is a rapid, ultrasensitive, highly specific, and readily available detection method which can be used as a valuable diagnostic tool for MTC infection in clinical settings.

## MATERIALS AND METHODS

### Ethical statement.

The study was approved by the Human Ethics Committee of the Guizhou Provincial Center for Disease Control and Prevention and complied with the Declaration of Helsinki. All data/isolates were analyzed anonymously.

### Materials and instruments.

Universal DNA isothermal amplification kits obtained from Bei-Jing HaiTaiZhengYuan. Co., Ltd. (Beijing, China). Modified Roche medium was provided by Zhuhai Baso Biotechnology Co., Ltd. (Zhuhai, China). Universal genomic DNA extraction kits were purchased from TaKaRa Biomedical Technology (Beijing) Co. Ltd. (Beijing, China). Malachite green reagents (MG) were purchased from Tian-Jin Huidexin Technology Development Co., Ltd. (Tianjin, China). AapCas12b nuclease (C2c1, 10 pmol/μL) purchased from Tian-Jin Huidexin Technology Development Co., Ltd. (Tianjin, China). Real-time fluorescence quantitative detector was obtained from Applied Biosystems (Waltham, MA, USA). Real-time turbidimeter (LA-500) was provided by Eiken Chemical Co., Ltd. (Tokyo, Japan). The conventional PCR thermal cycler was obtained from Hangzhou Bioer Technology Co., Ltd. (Hangzhou, China). The ChemiDoc MP imaging system obtained from Bio-Rad (Hercules, CA, USA). Benchtop high-speed refrigerated centrifuge (3-18K) obtained from Sigma (Germany). The biosafety cabinet (MSC-Advantage) provided by Thermo Fisher Scientific Co., Ltd. (Beijing, China).

### Design and synthesis of gRNA and MCDA primers.

In the MCDA reaction system, a total of 10 primers were used to recognize 10 different regions of the target, including two displacement primers (F1 and F2), two cross-primers (CP1 and CP2), and six amplification primers (D1, D2, C1, C2, R1, and R2). Next, these specific MCDA primers, targeting the *IS6110* sequence (GenBank ID: X17348.1), were designed according to the principle of MCDA amplification using the Primer Premier software (version 5.0). The designed primers were analyzed and screened by BLASTN software (basic local alignment search tool). Crucially, the gRNA sequence (composed of both crRNA and tracrRNA) which guides the Cas12b protein to perform *trans*-cleavage was designed according to the principles of CRISPR/Cas12b reaction. In the MCDA reaction system, the conventional CP1 or CP2 primers mainly consist of two recognition regions (target-dependent regions): a 5′-terminal region (20 to 24 bp) that is reverse-complementary to the DNA templates and a 3′-terminal region (16 to 22 bp) that is complementary to the DNA templates. Importantly, there is also a linker region (0 to 4 bp) in the middle of these two recognition regions, which was used as an ideal modification of the PAM site because it is independent of the target sequence. Thus, in our design, the CP1 primer was specifically modified with a new PAM site (TTTC) in the linker region (Fig. S1, Table S1). Details on the gRNA, MCDA primers, and ssDNA reporter (including locations, sequences and lengths, and PAM site) are presented in Fig. S1 and Table S1. In addition, the conventional MTC-LAMP and PCR assays established earlier in our study were implemented as comparison methods, and their sequences are also shown in Table S1. All high-performance liquid chromatography (HPLC)-purified primers and gRNA used in the experiments were synthesized by Tianyi-Huiyuan Biotech Co., Ltd. (Beijing, China).

### Preparation of DNA templates.

A total of 35 bacterial strains, including 14 MTC (MTB H37Rv, MTB H37Ra, M. bovis, M. bovis bacillus Calmette-Guérin vaccine strain, M. africanum, and 9 MTB isolates), 11 non-tuberculous mycobacteria (NTM), and 10 non-mycobacteria strains, were used to prepare DNA templates using the universal DNA extraction kits, and the extraction steps were performed according to the manufacturer's instructions. The genomic DNA extracted from MTB H37Rv (ATCC 27294) were diluted and prepared into serial dilutions (1 ng/μl, 100 pg/μL, 10 pg/μL, 1 pg/μL, 100 fg/μL, 10 fg/μL, 5 fg/μL, 1 fg/μL, and 500 ag/μL) using an ultramicro spectrophotometer (Thermo Fisher Scientific Co., Ltd., Beijing, China). The details of the bacterial strains used in this report, including names, sources, and detection results, are shown in Table S3.

A total of 90 clinical sputum samples were collected from the Guizhou Provincial Center for Disease Control and Prevention (including 63 from TB patients and 27 from non-TB patients). These TB patients were diagnosed based on clinical symptoms suggestive of active TB plus positive results by smear-staining microscopy (Ziehl-Neelsen staining) and/or GeneXpert MTB/RIF assay and/or mycobacterial culture. According to these diagnostic criteria, the sputum samples collected from confirmed TB cases were included in the TB group. Meanwhile, the sputum specimens from patients diagnosed with non-TB infectious diseases of the respiratory system were included in the non-TB group. Moreover, sputum samples of appropriate volume which met the detection requirements (usually >2 mL) were also screened in our study. Next, these sputum samples pretreated with 4% NaOH were equally divided into two halves (sections A and B). Section A was subjected to smear-staining microscopy (Ziehl-Neelsen staining) and cultured using modified Roche medium according to the manufacturer’s instructions (Zhuhai Baso Biotechnology Co., Ltd., Zhuhai, China). Section B was used to prepare DNA templates to evaluate the utility of the CRISPR-MCDA assay by comparing it with conventional PCR and LAMP assays. Briefly, the quick extraction steps for clinical samples were as follows. After the clinical sputum samples were liquefied (1 to 2 volumes of 4% NaOH), 1 mL of the liquefied sample was placed in a 1.5-mL centrifuge tube and centrifuged at 11,000 rpm for 5 min, and the supernatant was discarded; Then, when 1 mL of phosphate-buffered saline (PBS [pH 6.8 to 7.2]) had been added to resuspend the bacteria solution, the centrifuge tube was centrifuged again at 11,000 rpm for 5 min and the supernatant was discarded; Finally, 50 μL of DNA extraction solution (Ustar Biotechnology Co., Ltd., Hangzhou, China) was added to the centrifuge tube, which was placed in a heating block to boil for 8 min and centrifuged at 11,000 rpm for 2 min, and the supernatant was used as the DNA template (stored at −20°C). All centrifugation steps were performed under refrigerated conditions using a benchtop high-speed refrigerated centrifuge (3-18K). Moreover, the sputum samples processing was carried out in a biosafety cabinet (MSC-Advantage) to protect the health of laboratory personnel.

### MCDA pre-amplification.

The 25-μL amplification system for the MCDA experiments in our study consisted of the following: 12.5 μL 2× BF (amplification buffer); 1 μL 2.0 *Bst* DNA polymerase (8 U); 0.4 μM each of F1 and F2 primers (displacement primers); 0.8 μM each of C1, C2, R1, R2, D1, and D2 primers (amplification primers); 1.6 μM each of CP1 and CP2 primers (cross primers); 1 μL MG visualization reagent (only used in confirmation experiments); and 1 μL of DNA template extracted from pure cultures or 5 μL of DNA templates from clinical specimens; and then nuclease-free water was added to a volume of 25 μL. The reaction tube was incubated at 66°C for 50 min and then inactivated at 85°C for 5 min. In this experiment, 1 μL of DNA template of Klebsiella pneumoniae was used as a negative control and 1 μL of nuclease-free water was used as a blank control. Finally, amplification products were verified by a real-time turbidimeter, 1.5% agarose gel electrophoresis, or MG visualization indicator (added when premixing the reaction system).

### CRISPR/Cas12b-mediated *trans*-cleavage detection.

The Cas12b nucleases were pre-combined with gRNAs to form the Cas12b/gRNA complex, which was used to achieve *trans*-cleavage of the ssDNA reporters for MTC detection. That is, 1.5 μM Cas12b nuclease (final concentration: 150 nM) was pre-incubated with 2.2 μM gRNAs (final concentration: 220 nM) in 1× HuiDeXin buffer to form the Cas12b/gRNA (namely, ribonucleoprotein) complex. Subsequently, pre-assembled tubes were incubated at 37°C for 15 min, and the complexes were used immediately within 12 h and stored at 4°C. The CRISPR/Cas12b-gRNA-mediated *trans*-cleavage system (20 μL) was as composed of the following: 10 μL HuiDeXin buffer (2×), 5 μL Cas12b/gRNA complex, 1 μL ssDNA probe (5′-FAM-TTATTAT-BHQ1-3′, 100 μM), 2 μL MCDA amplicons, and 2 μL nuclease-free water. The *trans*-cleavage reaction was carried out at 48°C for 30 min, and the *trans*-cleavage results were detected by real-time fluorescence analysis and/or visualization detection under UV light (positive reaction was bright green, while negative reaction was colorless).

### Optimization of pre-amplification conditions for CRISPR-MCDA assay.

To obtain optimal detection efficiency, the pre-amplification temperature of the MCDA assay was optimized by performing it at different temperatures (62 to 69°C, 1-°C intervals), and the results were monitored using a real-time turbidimeter. Meanwhile, a real-time fluorescence assay was also used to verify pre-amplification results after completion of the Cas12b/gRNA-mediated *trans*-cleavage reaction. One μL of MTB H37Rv (ATCC 27294) genomic DNA (1 ng/μL) was used as the amplification template. Moreover, to confirm the optimal pre-amplification time for the CRISPR/Cas12b-MCDA assay, 1 μL of each dilution of the MTB H37Rv genome (1 ng/μL, 100 pg/μL, 10 pg/μL, 1 pg/μL, 100 fg/μL, 10 fg/μL, 5 fg/μL, 1 fg/μL, and 500 ag/μL) was amplified individually by setting different durations (25 to 50 min with 5-min intervals). The *trans*-cleavage results were verified by a real-time fluorescence detector and visualization detection under UV light. The CRISPR/Cas12b-MCDA assays were conducted as previously described in this report.

### Optimization of the *trans*-cleavage time for CRISPR-MCDA assay.

To achieve rapid detection, the optimal *trans*-cleavage time for real-time fluorescence analysis and visualization detection was explored by setting different durations (5 to 30 min, 5-min intervals). One μL of each dilution of the MTB H37Rv genome (1 ng/μL, 100 pg/μL, 10 pg/μL, 1 pg/μL, 100 fg/μL, 10 fg/μL, 5 fg/μL, 1 fg/μL, and 500 ag/μL) was used as the templates according to the optimal pre-amplification conditions. Then, CRISPR-MCDA assays were performed according to the MCDA reaction and Cas12b-mediated *trans*-cleavage detection, and the results were validated by real-time fluorescence analysis and visualization (under UV light).

### Conventional MTC-LAMP and PCRs.

In our current report, the conventional MTC-LAMP and PCR assays were implemented as comparison methods to verify their analytical sensitivity and detection ability for sputum specimens. The reaction system of MTC-LAMP test (25 μL) was as follows: 12.5 μL 2× BF (reaction buffer), 1 μL 2.0 *Bst* DNA polymerase, 1.6 μM (each) FIP and BIP primers, 0.8 μM (each) LF and LB primers, 0.4 μM (each) F3 and B3 primers, 1 μL of DNA template extracted from pure culture or 5 μL of DNA template from samples, and nuclease-free water added to a volume of 25 μL. The MTC-LAMP assays were performed using the previously optimized reaction conditions (66°C, 40 min), and the amplification results were tested by 1.5% agarose gel electrophoresis and real-time turbidity (threshold value = 0.1, turbidity of >0.1 was judged as positive).

In addition, the MTC-PCR assays were carried out according to common detection procedures, and the reaction mixtures (25 μL) consisted of 12.5 μL 2× *Taq* Master Mix (CoWin Biosciences Co., Ltd. Beijing, China), 0.2 μM *IS6110*-F primer, 0.2 μM *IS6110*-R primer, 1 μL of DNA template extracted from pure culture or 5 μL of DNA template from sample, and nuclease-free water added to a volume of 25 μL. The PCR-premixed solutions were denatured at 94°C for 2 min and 30 reaction cycles were performed. The cycles consisted of denaturation at 94°C (30 s), annealing at 59°C (30 s), and primer extension at 72°C (30 s). The final extension time was set at 2 min. Amplification results (amplicon size: 439 bp) were validated by 1.5% agarose gel electrophoresis with GelRed staining and subsequently visualized by the ChemiDoc MP imaging system.

### Sensitivity and specificity of MTC-CRISPR-MCDA assays.

To explore the limit of detection of the CRISPR-MCDA assay, each dilution of MTB genomic DNA (1 ng/μL, 100 pg/μL, 10 pg/μL, 1 pg/μL, 100 fg/μL, 10 fg/μL, 5 fg/μL, 1 fg/μL, and 500 ag/μL) was tested, and the test was performed using a defined number of replicates (usually 20 per dilution). The LoD of CRISPR-MCDA was defined as the lowest concentration of genomic DNA that was able to detect MTB in ≥95% of the tests performed in this study by serial dilution assays (17). The *trans*-cleavage results were verified using a real-time fluorescence detector and visual detection (under UV light).

These genomic DNAs extracted from 35 bacteria strains, including 5 MTC reference strains (MTB H37Rv, MTB H37Ra, M. bovis, M. bovis bacillus Calmette-Guérin vaccine strain, and M. africanum), 9 MTB isolates, 11 NTM strains, and 10 non-mycobacteria strains, were used to evaluate the specificity of the CRISPR-MCDA assay according to the optimal reaction conditions. The specificity test was performed in triplicate, and the results were verified using real-time fluorescence analysis and visualization validation under UV light.

### Evaluation of the practicability of MTC-CRISPR-MCDA in clinical samples.

To evaluate the utility of MTC-CRISPR-MCDA for testing of clinical samples, the DNA templates extracted from 90 clinical sputum samples (63 from TB patients and 27 from non-TB patients) were tested by MTC-CRISPR-MCDA, conventional LAMP, and PCR assays. Meanwhile, the sputum samples were also subjected to traditional smear-staining microscopy and mycobacteria culture. Next, the applicability of MTC-CRISPR-MCDA for clinical sputum samples was analyzed and evaluated by comparing it with smear-staining microscopy, culture, MTC-LAMP, and MTC-PCR methods. The MTC-CRISPR-MCDA assays were performed according to optimal reaction conditions, and these methods (smear-staining microscopy, culture, MTC-PCR, and MTC-LAMP tests) were implemented as described above.

### Data availability statement.

All data sets generated for this research are contained in the manuscript.
