# Supplementary material for: Development of CRISPR/Cas12b-Based Multiple Cross Displacement Amplification Technique for the Detection of Mycobacterium tuberculosis Complex in Clinical Settings
Source: Microbiol Spectr. 2023 Mar 28;11(2):e03475-22. doi: 10.1128/spectrum.03475-22 (PMC10100757; doi:10.1128/spectrum.03475-22)
Supplement: Supplemental file 1 — Fig. S1 to S10 and Tables S1 to S3. Download spectrum.03475-22-s0001.pdf, PDF file, 1.5 MB [file spectrum.03475-22-s0001.pdf]

Supplementary Material

Development of CRISPR/Cas12b-Based Multiple Cross Displacement Amplification Technique for the Detection of *Mycobacterium tuberculosis* Complex in Clinical Settings

Xinggui Yang [a], Junfei Huang [a], Yijiang Chen [a], Xia Ying [a], Qinqin Tan [a], Xu Chen [b], Xiaoyan Zeng [b], Shiguang Lei [a], Yi Wang [c, *], Shijun Li [a, *]

[a] Guizhou Provincial Center for Disease Control and Prevention, Guiyang, 550004, Guizhou, P.R. China.

[b] The Second Affiliated Hospital, Guizhou University of Traditional Chinese Medicine, Guiyang, 550003, Guizhou, P.R. China.

[c] Experimental Research Center, Capital Institute of Pediatrics, Beijing 100020, P.R. China.

Address correspondence to Shijun Li (zjumedjun@163.com) or Yi Wang (wildwolf0101@163.com).

Supplementary Figures

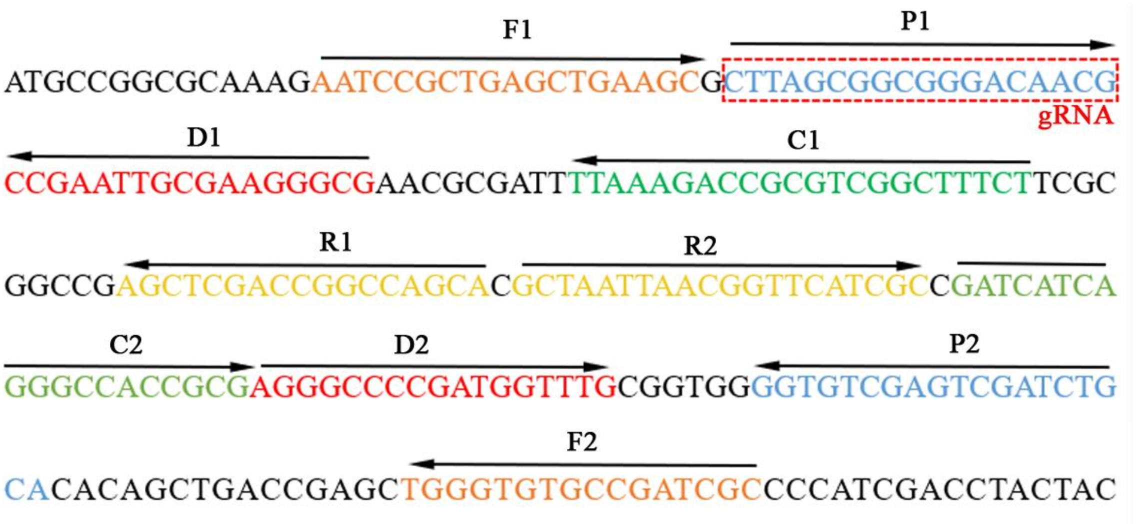

Supplementary Fig. S1 The location and sequence of MCDA primers and gRNA used in CRISPR-MCDA assay. Primer sequences were shown in colored font, and the gRNA was displayed in red dashed boxes. The direction of arrows indicated the primer from 5′ to 3′. CRISPR: clustered regularly interspaced short palindromic repeats; MCDA: multiple cross displacement amplification; gRNA: guide RNA.

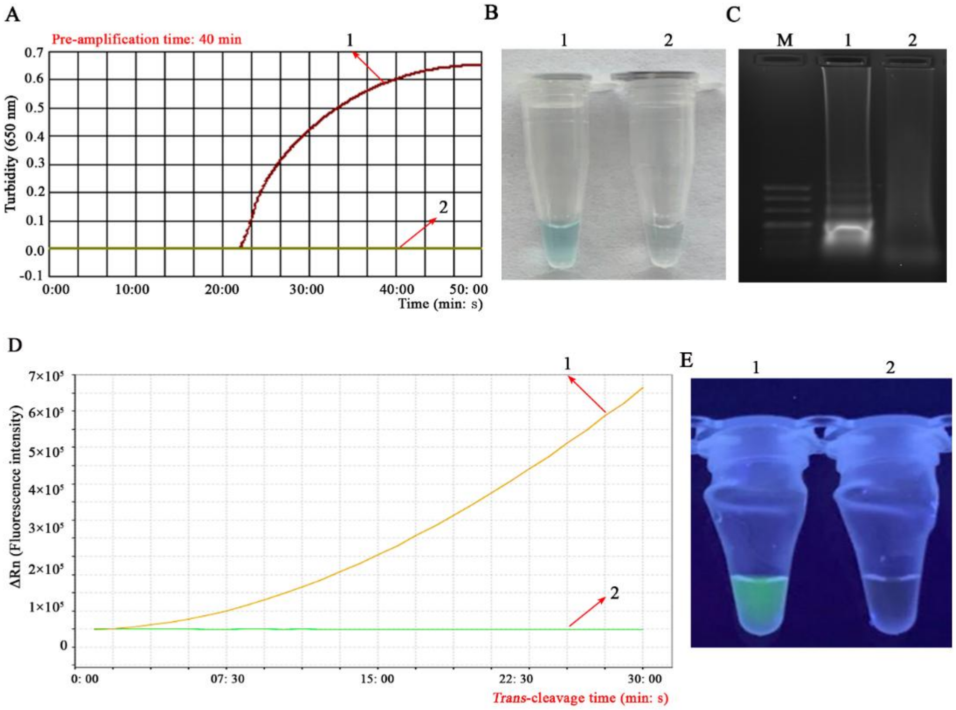

Supplementary Fig. S2 Confirmative tests for MCDA pre-amplification and Cas12b/gRNA-mediated *trans*-cleavage detection in CRISPR-MCDA assays. There were two main steps, including the verification of the MCDA pre-amplification products (**A, B, and C**) and the confirmation of the *trans*-cleavage detection (**D and E**). MCDA amplicons were verified by real-time turbidimeter (**A**), MG visual indicator (**B**), and 1.5% agarose gel electrophoresis (**C**). The Cas12b/gRNA-mediated *trans*-cleavage was then confirmed by real-time fluorescence analysis (**D**) and visualization detection (**E**). Turbidity (**A1**)/tube (**B1**)/lane (**C1**)/fluorescence signal (**D1**) /fluorescence vessel (**E1**): positive reactions of CRISPR-MCDA assay; Turbidity (**A2**)/tube (**B2**)/lane (**C2**)/fluorescence signal (**D2**) /fluorescence vessel (**E2**): blank control of CRISPR-MCDA assay (nuclease-free water). Line **M**: 100 bp DNA ladder. MG: malachite green; CRISPR/Cas12b: clustered regularly interspaced short palindromic repeats (CRISPR)/CRISPR-associated 12b protein; MCDA: multiple cross displacement amplification; gRNA: guide RNA.

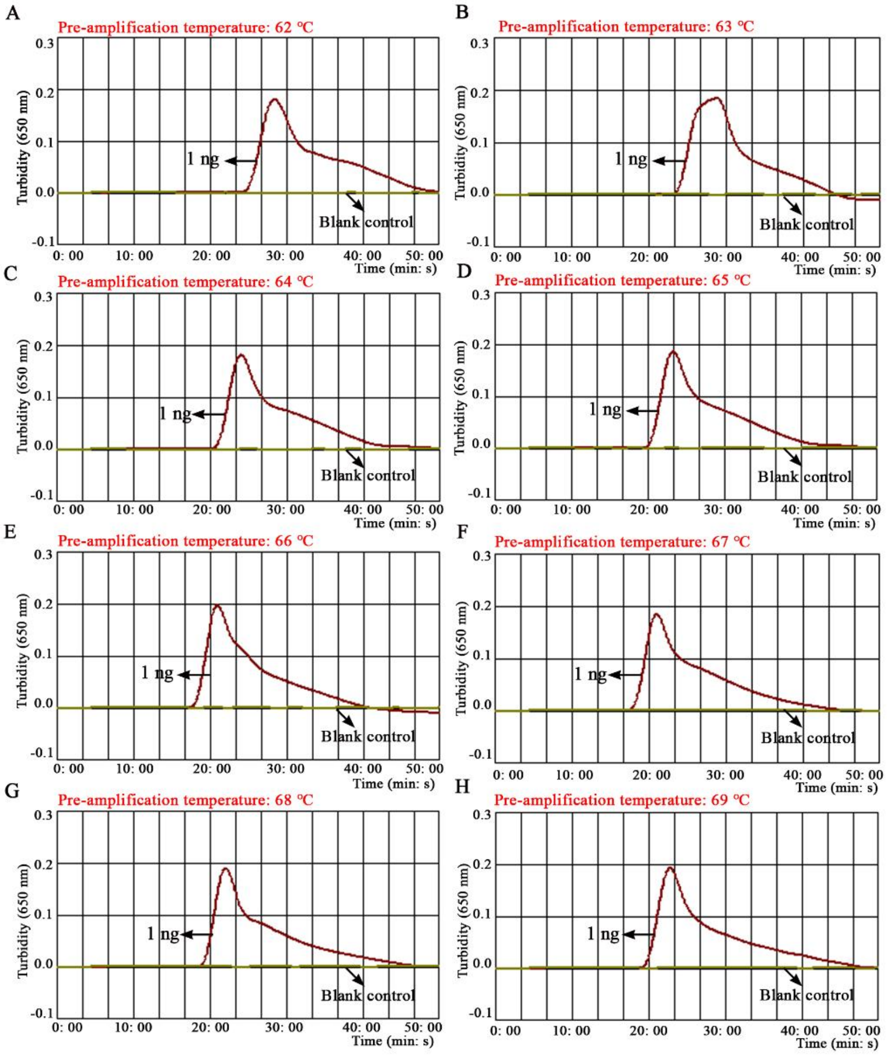

Supplementary Fig. S3 The optimal pre-amplification temperature of the CRISPR-MCDA assay by real-time turbidity. The incubation results of the MCDA pre-amplification were monitored by a real-time turbidimeter, and the turbidity threshold value was 0.1 and then a turbidity value > 0.1 is considered as positive amplification. A total of eight kinetic curves (**A-H**) were obtained at different amplification temperatures (62 to 69 °C with 1 °C intervals). These curves showed that the CRISPR-MCDA assay has better amplification efficiency at temperatures ranging from 65 to 68 °C (**D, E, F,**

and G), with the optimal efficiency at 66 °C (**E**). CRISPR: clustered regularly interspaced short palindromic repeats; MCDA: multiple cross displacement amplification.

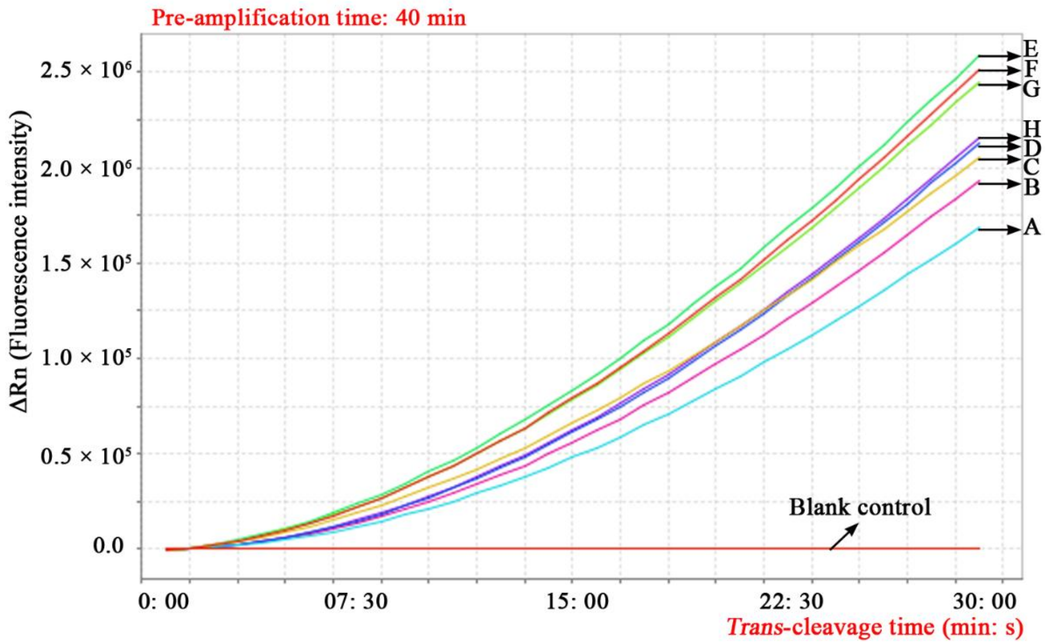

Supplementary Fig. S4 The optimal pre-amplification temperature of CRISPR-MCDA assay by Cas12b/gRNA-mediated *trans*-cleavage detection. Real-time fluorescence detection was used for reporting *trans*-cleavage results. The fluorescence signals (**A-H**) indicated that the pre-amplification temperature was 62, 63, 64, 65, 66, 67, 68, and 69 ℃, respectively. These signals demonstrated that the CRISPR-MCDA assays have the optimal amplification efficiency at 66 °C (**E**), and the results are consistent with real-time turbidity. CRISPR/Cas12b: clustered regularly interspaced short palindromic repeats (CRISPR)/CRISPR-associated 12b protein; MCDA: multiple cross displacement amplification; gRNA: guide RNA.

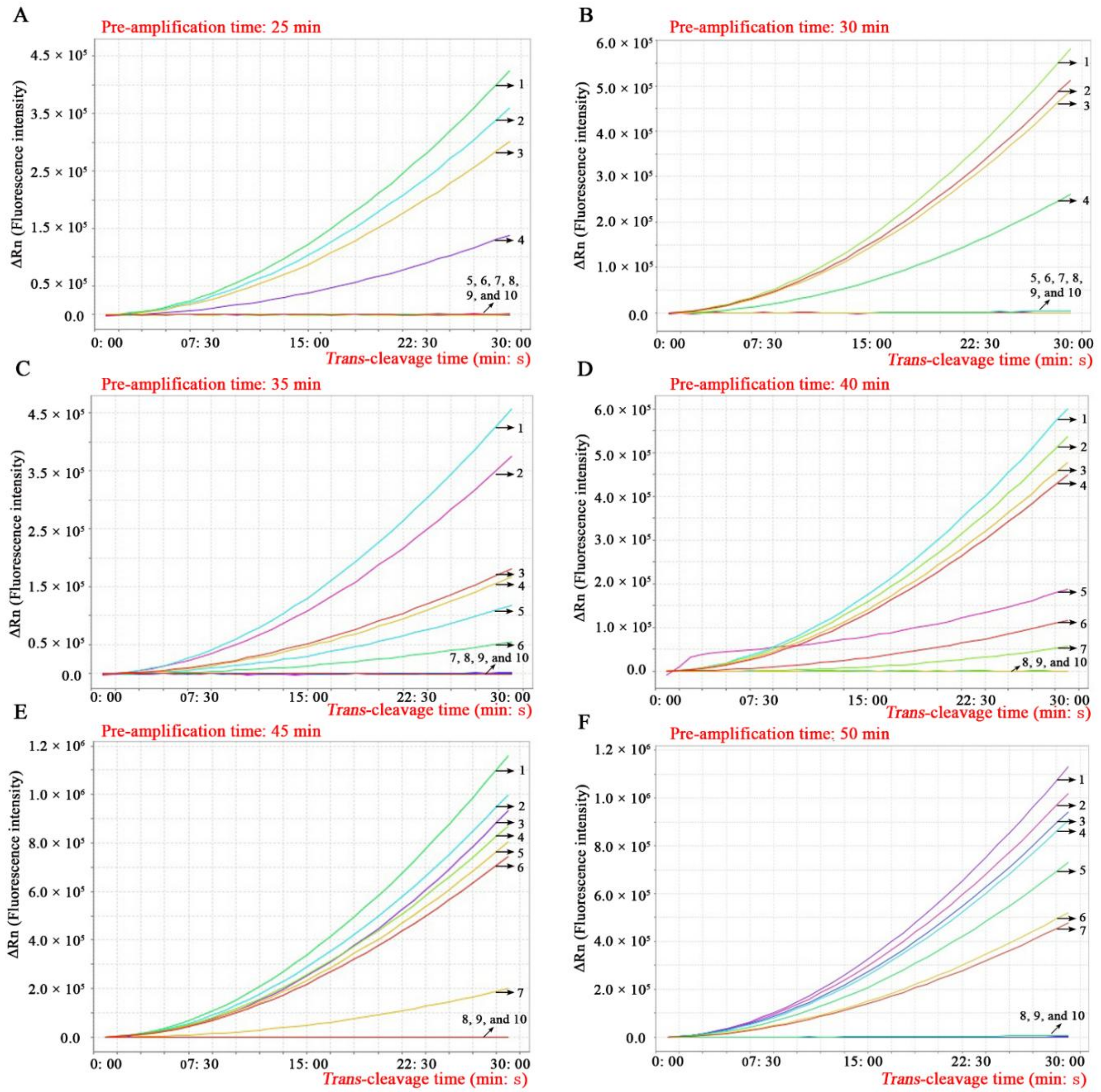

Supplementary Fig. S5 The optimal pre-amplification time of CRISPR-MCDA assay using real-time fluorescence analysis. Optimal pre-amplification time was confirmed using real-time fluorescence analysis by detection of each diluent of MTB H37Rv genome (1 ng, 100 pg, 10 pg, 1 pg, 100 fg, 10 fg, 5 fg, 1 fg, and 500 ag/µl). Six fluorescence plots (**A-F**) were generated corresponding to 25, 30, 35, 40, 45, and 50 min, respectively. Fluorescence signals **1-9** (**A-F**) correspond to DNA template of MTB

H37Rv from 1 ng/µl to 500 ag/µl. Fluorescence signals **10** (**A-F**) correspond to blank control (nuclease-free water). The lowest concentration (5 fg/µl) of serial dilutions of MTB genomic DNA that could be detected by the CRISPR-MCDA assay was consistent when the reaction time was from 40 to 50 min (**D, E, and F**). CRISPR: clustered regularly interspaced short palindromic repeats; MCDA: multiple cross displacement amplification; gRNA: guide RNA; MTB: *Mycobacterium tuberculosis.*

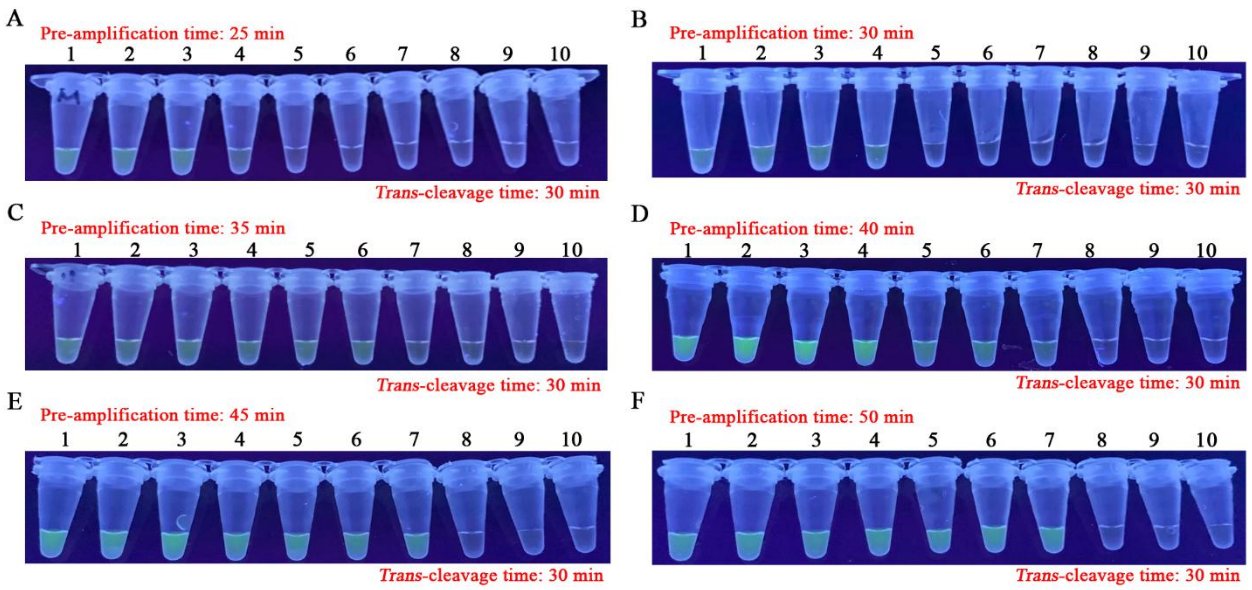

Supplementary Fig. S6 The optimal pre-amplification time of CRISPR-MCDA assay using visualization analysis (under UV light). Optimal pre-amplification time was confirmed using visualization detection by test of each diluent of MTB H37Rv genome (1 ng, 100 pg, 10 pg, 1 pg, 100 fg, 10 fg, 5 fg, 1 fg, and 500 ag/μl). Six visual drawings (**A-F**) were displayed corresponding to 25, 30, 35, 40, 45, and 50 min, respectively. Tubes **1-9** (**A-F**) correspond to DNA template of MTB H37Rv from 1 ng/μl to 500 ag/μl. Tubes **10** (**A-F**) correspond to blank control (nuclease-free water). The lowest concentration (5 fg/μl) of serial dilutions of MTB genomic DNA that could be detected by the visualization analysis was consistent when the reaction time was from 40 to 50 min (**D, E, and F**). CRISPR: clustered regularly interspaced short palindromic repeats; MCDA: multiple cross displacement amplification; gRNA: guide RNA; MTB: *Mycobacterium tuberculosis.*

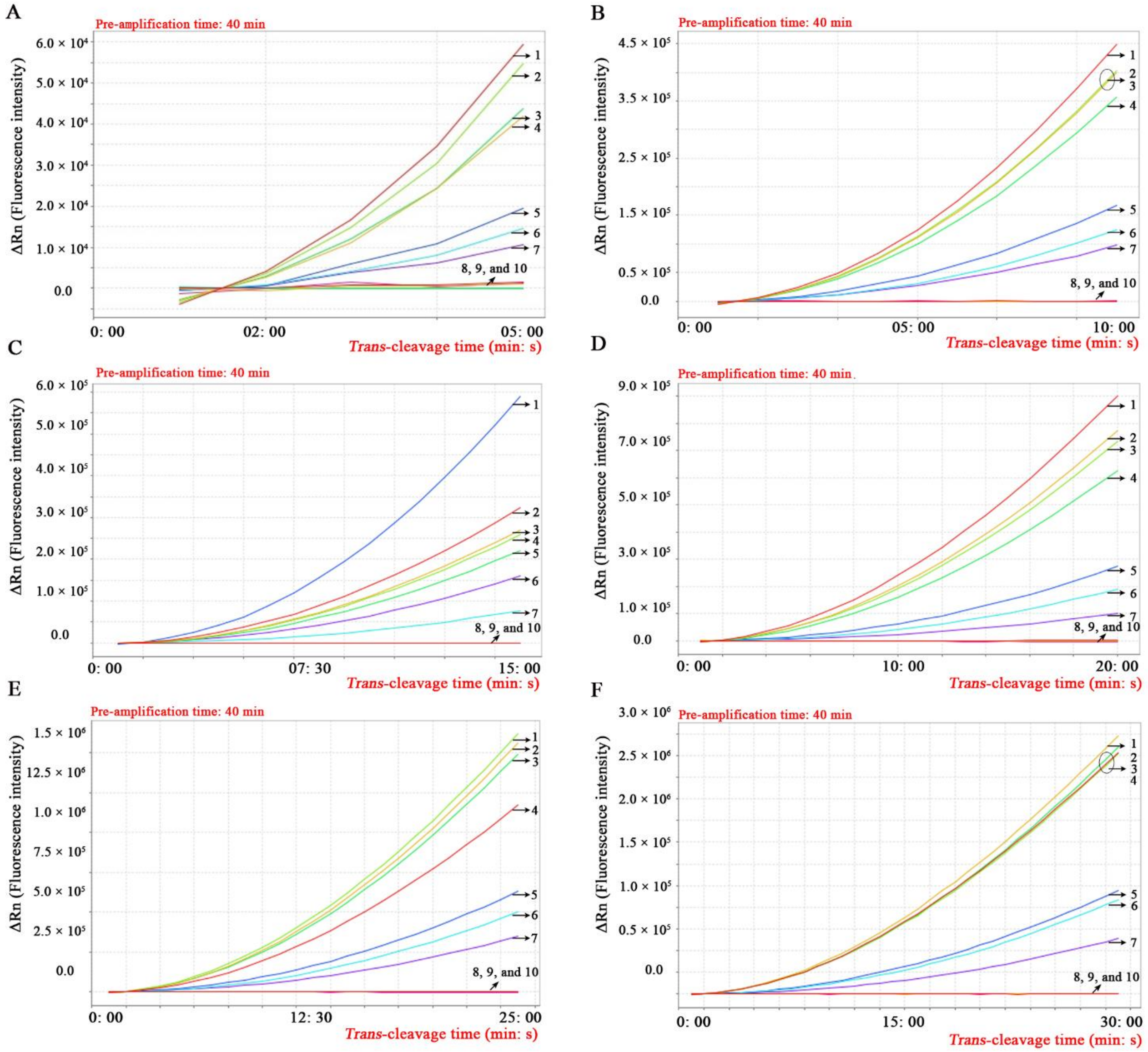

Supplementary Fig. S7 The optimal *trans*-cleavage time of the CRISPR-MCDA assay using real-time fluorescence analysis. One microliter of each dilution of MTB H37Rv genome (1 ng, 100 pg, 10 pg, 1 pg, 100 fg, 10 fg, 5 fg, 1 fg, and 500 ag/µl) was used as templates to optimize the *trans*-cleavage time. Six fluorescence plots (**A-F**) were generated corresponding to 5, 10, 15, 20, 25, and 30 min, respectively. Fluorescence signals **1-9** (**A-F**) correspond to DNA template of MTB H37Rv from 1 ng/µl to 500 ag/µl. Fluorescence signals **10** (**A-F**) correspond to blank control (nuclease-free water).

The lowest concentration (5 fg/μl) of serial dilutions of MTB genomic DNA that could be detected by the real-time fluorescence analysis was consistent when the *trans*-cleavage time was from 5 to 30 min (**D, E, and F**). CRISPR: clustered regularly interspaced short palindromic repeats (CRISPR); MCDA: multiple cross displacement amplification; gRNA: guide RNA; MTB: *Mycobacterium tuberculosis.*

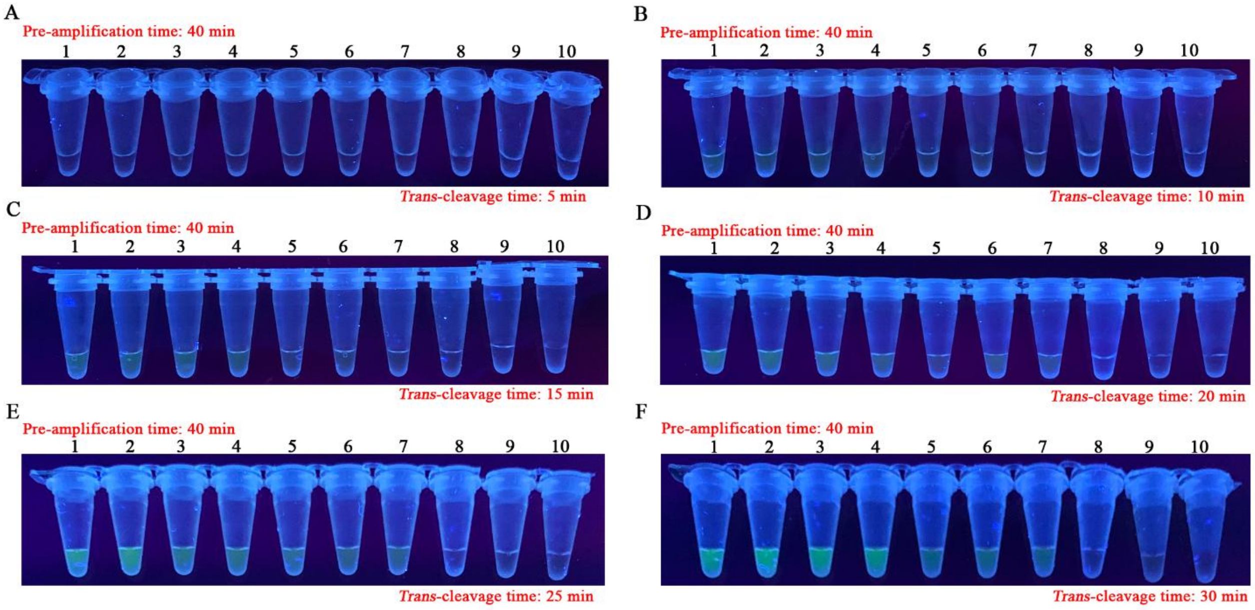

Supplementary Fig. S8 The optimal *trans*-cleavage time of the CRISPR-MCDA assay using visualization analysis (under UV light). One microliter of each dilution of MTB H37Rv genome (1 ng, 100 pg, 10 pg, 1 pg, 100 fg, 10 fg, 5 fg, 1 fg, and 500 ag/μl) was used as templates to optimize the *trans*-cleavage time. Six visual pictures (**A-F**) were showed corresponding to 5, 10, 15, 20, 25, and 30 min, respectively. Tubes **1-9** (**A-F**) correspond to DNA template of MTB H37Rv from 1 ng/μl to 500 ag/μl. Tubes **10** (**A-F**) correspond to blank control (nuclease-free water). The lowest concentration (5 fg/μl) of serial dilutions of MTB genomic DNA that could be detected by visualization detection was consistent when the *trans*-cleavage time was from 25 to 30 min (**D, E, and F**). CRISPR: clustered regularly interspaced short palindromic repeats; MCDA: multiple cross displacement amplification; MTB: *Mycobacterium tuberculosis.*

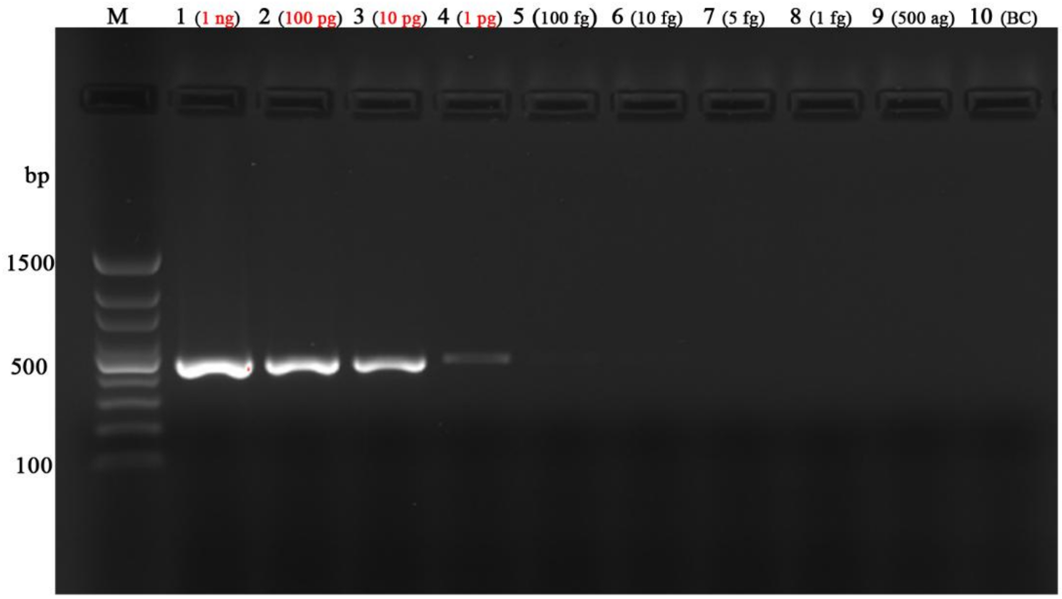

Supplementary Fig. S9 Analytical sensitivity for the *IS6110*-PCR assay. The *IS6110*-PCR assays were performed according to conventional PCR reactions as described above, and each diluent (1 ng, 100 pg, 10 pg, 1 pg, 100 fg, 10 fg, 5 fg, 1 fg, and 500 ag/μl) of the MTB genomic DNA (H37Rv, ATCC 27294) was used as an amplification template. PCR amplicons (439 bp) were verified by 1.5% agarose gel electrophoresis with GelRed staining and subsequently visualized by ChemiDoc MP imaging system. Lanes **1-9** correspond to DNA template of MTB H37Rv from 1 ng/μl to 500 ag/μl. Lane **10** corresponds to blank control (nuclease-free water). Line **M**: 100 bp DNA ladder. PCR: polymerase chain reaction; MTB: *Mycobacterium tuberculosis.*

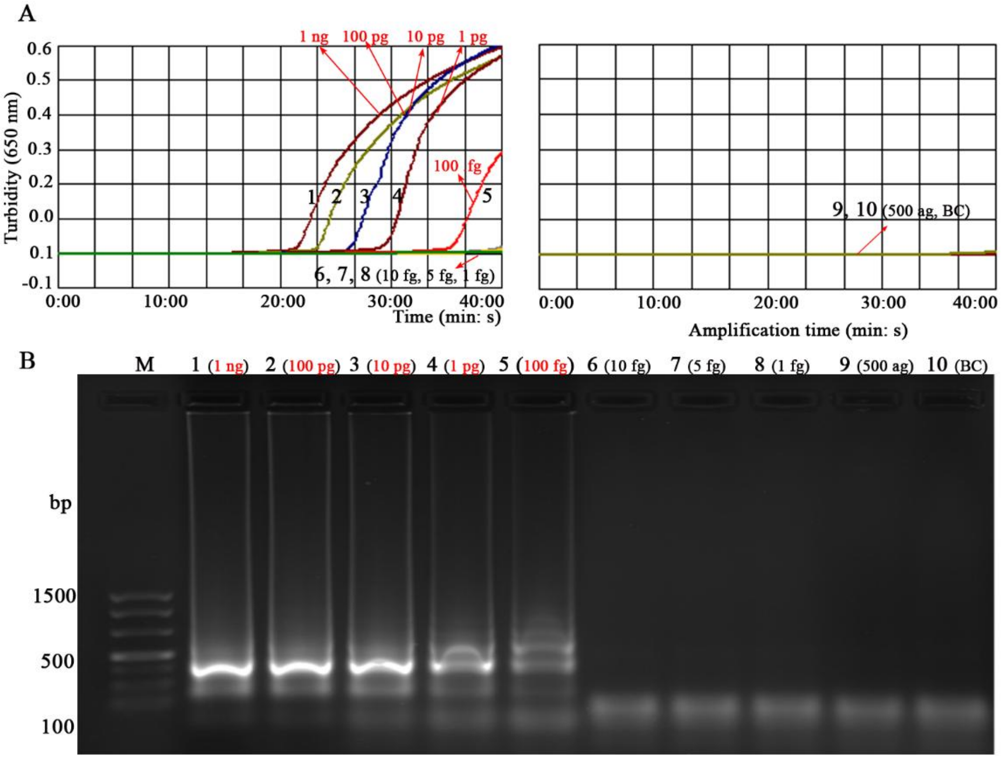

Supplementary Fig. S10 Analytical sensitivity for the *IS6110*-LAMP assay. The *IS6110*-LAMP assays were performed according to conventional LAMP reactions as described above, and each diluent (1 ng, 100 pg, 10 pg, 1 pg, 100 fg, 10 fg, 5 fg, 1 fg, and 500 ag/μl) of the MTB genomic DNA (H37Rv, ATCC 27294) was used as an amplification template. LAMP amplicons were verified by real-time turbidity and 1.5% agarose gel electrophoresis with GelRed staining (ChemiDoc MP imaging system). Turbidities (**A**)/lanes (**B**) **1-9** correspond to DNA template of MTB H37Rv from 1 ng/μl to 500 ag/μl. Turbidity (**A**)/lanes (**B**) **10** corresponds to blank control (nuclease-free water). Line **M**: 100 bp DNA ladder; LAMP: loop-mediated isothermal amplification; MTB: *Mycobacterium tuberculosis.*

Supplementary Tables

Supplementary Table S1. The primers used in the current study

**Primers/gRNA** [a]	**Sequences and modifications** [b]	**Length** [c]
IS6110-F1	5'-AATCCGCTGAGCTGAAGC-3'	18 nt
IS6110-F2	5'-GCGATCGGCACACCCA-3'	16 nt
IS6110-CP1	5'-AGAAAGCCGACGCGGTCTTTAATTTCCTTAGCGGCGGGACAACG-3'	44 mer
IS6110-CP2	5'-GATCATCAGGGCCACCGCGTGCAGATCGACTCGACACC-3'	38 mer
IS6110-C1	5'-AGAAAGCCGACGCGGTCTTTAA-3'	22 nt
IS6110-C2	5'-GATCATCAGGGCCACCGCG-3'	19 nt
IS6110-D1	5'-CGCCCTTCGCAATTCGG-3'	21 nt
IS6110-D2	5'-AGGGCCCCGATGGTTTG-3'	17 nt
IS6110-R1	5'-TGCTGGCCGGTCGAGCT-3'	17 nt
IS6110-R2	5'-GCTAATTAACGGTTCATCGC-3'	20 nt
gRNA	5'-<br>GUCUAGAGGACAGAAUUUUUCAACGGGUGUGCCAAUGGCCACUUUCCA<br>GGUGGCAAAGCCCGUUGAGCUUCUCAAAUCUGAGAAGUGGCACCUUAG<br>CGGCGGGACAACG-3'	109 mer
Probe	5'-FAM-TTATTAT-BHQ1-3'	7 nt
IS6110-F3	5′-AGAGATCCGCGGTCAGC-3′	17 nt
IS6110-B3	5′-ATTCGGCGTTGTCCCG-3′	16 nt
IS6110-FIP	5′-CGCGCAGCCAACACCAAGTACGATTCGGAGTGGGCAG-3′	37 mer
IS6110-BIP	5′-GAGACGGTGCGTAAGTGGGTTCAGCGGATTCTTCGGTC-3′	38 mer
IS6110-LF	5′-GGCGACCTCACTGATC-3′	16 nt
IS6110-LB	5′-AGGCGCAGGTCGATG-3′	15 nt
IS6110-F	TCGAACGGCTGATGACCAAA	20 nt
IS6110-R	GTCATAGGAGCTTCCGACCG	20 nt

[a] gRNA: guide RNA; MCDA primers included *IS6110*-F1, -F2, -CP1, -CP2, -C1, -C2, -D1, -D2, -R1, and -R2; LAMP primers contained *IS6110*-F3, -B3, -FIP, -BIP, -LF, and -LB; PCR primers consisted of *IS6110*-F and -R.

[b] The CP1 primer was modified with a PAM site (TTTC) in the linker region.

[c] mer: monomeric unit; nt: nucleotide.

Supplementary Table S2. Comparison of CRISPR-MCDA with the MTC-PCR, MTC-LAMP, SSM, and Culture assays in the current study

Methods	CRISPR-MCDA[a]	MTC-PCR[b]	MTC-LAMP[c]	SSM[d]	Culture
Target sequence	*IS6110*	*IS6110*	*IS6110*	/	/
DNA preparation	Yes	Yes	Yes	/	/
Amplification assay	MCDA	PCR	LAMP	/	/
Limit of detection	5 fg/μl	1000 fg/μl	100 fg/μl	/	/
Detection sensitivity	80.95%	76.19%	73.02%	73.02%	57.14%
Detection specificity	100%	100%	100%	100%	100%
Quantitative	No	No	No	No	No
Detection time	70 min/ 95 min	110 min	105 min/ 70 min	120 min	4~8 weeks
Verification methods	Fluorescence/ VD	AGE	AGE/ Turbidity	Microscopy	Microscopy

[a] CRISPR: clustered regularly interspaced short palindromic repeats; MCDA: multiple cross displacement amplification; VD: visualization detection.

[b] MTC: *Mycobacterium tuberculosis* complex; PCR: polymerase chain reaction; AGE: agarose gel electrophoresis.

[c] LAMP: loop-mediated isothermal amplification.

[d] SSM: sputum smear microscopy.

Supplementary Table S3. Bacterial strains used in the current study

Bacteria [a]	Strain no. (source of strain) [b]	No. of strains	CRISPR-MCDA results [c]
MTB	H37Rv (ATCC 27294)	1	P
	H37Ra (ATCC 25177)	1	P
	isolated strains (GZCDC)	9	P
Mycobacterium bovis	ATCC 19210	1	P
Bacillus Calmette-Guerin	vaccine strain	1	P
Mycobacterium africanum	ATCC 25420	1	P
Mycobacterium smegmatis	ATCC 19420	1	N
Mycobacterium nonchromogenicum	ATCC 19530	1	N
Mycobacterium aichiense	ATCC 27280	1	N
Mycobacterium neoaurum	ATCC 25795	1	N
Mycobacterium scrofulaceum	ATCC 19981	1	N
Mycobacterium xenopi	ATCC 19250	1	N
Mycobacterium ulcerans	ATCC 19423	1	N
Mycobacterium malmoense	ATCC 29571	1	N
Mycobacterium abscessus	ATCC 19977	1	N
Mycobacterium kansassi	ATCC 12478	1	N
Mycobacterium vaccae	ATCC 15483	1	N
Brucella melitensis	M5 (vaccine strain)	1	N
Klebsiella pneumoniae	isolated strains (GZCDC)	1	N
Pseudomonas aeruginosa	isolated strains (GZCDC)	1	N
Haemophilus influenzae	isolated strains (GZCDC)	1	N
Streptococcus pneumoniae	isolated strains (GZCDC)	1	N
Staphylococcus aureus	isolated strains (GZCDC)	1	N
Shigella sonnei	isolated strains (GZCDC)	1	N
Bacillus anthracis	isolated strains (GZCDC)	1	N
Streptococcus suis	isolated strains (GZCDC)	1	N
Salmonella spp.	isolated strains (GZCDC)	1	N
Total		35	

[a] MTB: *Mycobacterium tuberculosis.*

[b] ATCC: American Type Culture Collection; GZCDC: Guizhou Provincial Center for Disease Control and Prevention.

[c] P: Positive; N: Negative.
